# Chromatin accessibility of primary human cancers ties regional mutational processes and signatures with tissues of origin

**DOI:** 10.1371/journal.pcbi.1010393

**Published:** 2022-08-10

**Authors:** Oliver Ocsenas, Jüri Reimand

**Affiliations:** 1 Computational Biology Program, Ontario Institute for Cancer Research, Toronto, Canada; 2 Department of Medical Biophysics, University of Toronto, Toronto, Canada; 3 Department of Molecular Genetics, University of Toronto, Toronto, Canada; Rutgers University, UNITED STATES

## Abstract

Somatic mutations in cancer genomes are associated with DNA replication timing (RT) and chromatin accessibility (CA), however these observations are based on normal tissues and cell lines while primary cancer epigenomes remain uncharacterised. Here we use machine learning to model megabase-scale mutation burden in 2,500 whole cancer genomes and 17 cancer types via a compendium of 900 CA and RT profiles covering primary cancers, normal tissues, and cell lines. CA profiles of primary cancers, rather than those of normal tissues, are most predictive of regional mutagenesis in most cancer types. Feature prioritisation shows that the epigenomes of matching cancer types and organ systems are often the strongest predictors of regional mutation burden, highlighting disease-specific associations of mutational processes. The genomic distributions of mutational signatures are also shaped by the epigenomes of matched cancer and tissue types, with SBS5/40, carcinogenic and unknown signatures most accurately predicted by our models. In contrast, fewer associations of RT and regional mutagenesis are found. Lastly, the models highlight genomic regions with overrepresented mutations that dramatically exceed epigenome-derived expectations and show a pan-cancer convergence to genes and pathways involved in development and oncogenesis, indicating the potential of this approach for coding and non-coding driver discovery. The association of regional mutational processes with the epigenomes of primary cancers suggests that the landscape of passenger mutations is predominantly shaped by the epigenomes of cancer cells after oncogenic transformation.

## Author summary

Cancer cells harbour thousands of somatic mutations that have complex genomic distributions. A few so-called driver mutations cause cancer by unlocking oncogenic pathways, while most mutations are functionally neutral passengers. Passenger mutations are caused by various mutational processes of aging, carcinogens, and errors in DNA repair. Therefore, passengers represent a footprint of cancer evolution that is informative of tumor development and exposures. The distribution of passenger mutations also associates with epigenetics, including chromatin accessibility and DNA replication timing. Here we use machine learning to show that mutational processes and signatures in cancer genomes associate with the epigenetic profiles of cancer cells, rather than normal tissues and common cell lines that have been indicated previously. This suggests that many passenger mutations occur after normal cells have transformed to cancer cells and acquired their epigenetic properties. These interactions are highly tissue-specific, as the epigenomes of specific cancer types are the strongest predictors of mutational processes in these cancer types. Our integrative models also indicate known and potential cancer driver mutations in the genomic regions where epigenetics does not fully explain the overrepresented mutations. Thus, our study characterises the complex interplay of mutational processes and genome function in cancer.

## Introduction

The cancer genome is a footprint of its evolution and molecular environment that is shaped by somatic mutations such as single nucleotide variants (SNVs) and structural alterations [1,2]. Cancer initiation and progression are caused by a small number of driver mutations that provide cells with selective advantages [3–5], however most mutations are functionally neutral passengers that are caused by various mutational processes [6–8]. Somatic mutations also occur in normal tissues and are frequently observed in known cancer genes [9,10]. Thus, we need to understand mutational processes to decipher tumor etiology and evolution and better characterise driver mutations.

Mutational processes act at different scales of the cancer genome [11,12]. Single base substitution (SBS) signatures affect certain trinucleotide context of the genome and are associated with aging, carcinogen exposures, defects in DNA repair pathways, and cancer therapies [6,13]. At a 100-nucleotide resolution, local mutational processes disproportionately affect certain non-coding genomic elements such as transcription start sites and binding sites of gene-regulatory proteins such as CTCF [14–16]. At the regional, megabase-scale resolution of the genome, mutation burden correlates with DNA replication timing (RT), chromatin accessibility (CA) and transcriptional activity [17–19]. Early-replicating, transcriptionally active regions of open chromatin have fewer mutations than late-replicating, passive regions of heterochromatin, potentially due to increased error rates and decreased mismatch repair later in DNA replication [20–23]. SBS signatures [24] as well as other classes of somatic mutations such as chromosomal rearrangements are distributed asymmetrically with respect to DNA replication origins and timing [25]. Regional mutation burden is associated with epigenetic information of related normal cells, suggesting that cells of cancer origin contribute to somatic variation [26] and allowing classification of cancers of unknown origin [27]. DNA methylation also correlates with mutational processes and DNA replication in cancer genomes [28]. Recent work has shown that mutational processes interact with three-dimensional chromatin and the boundaries of topologically associating domains [29]. However, the precise molecular mechanisms of regional mutational processes in various cancer types remain incompletely understood. In particular, cell lines and normal tissues have been used to associate chromatin and mutational processes in cancer, while the epigenetic landscapes of primary human cancers remain unexplored.

Here we studied cancer epigenomes as the predictors of regional mutagenesis in thousands of whole cancer genomes using a diverse collection of CA and RT profiles representing human cancers, normal tissues, and cell lines. CA profiles of primary human cancer samples, rather than those of normal tissues and common cell lines, show the strongest tissue-specific associations with regional mutagenesis and mutational signatures in most cancer types. Specific genomic regions where the observed mutation counts significantly exceed the epigenome-informed predictions show a pan-cancer convergence to cancer genes and developmental pathways. These results underline the spatial complexity of regional mutagenesis in cancer genomes and highlight epigenome-informed avenues to discover driver mutations.

## Results

### Chromatin accessibility profiles of primary cancers are stronger predictors of regional mutagenesis

**Fig 1:**
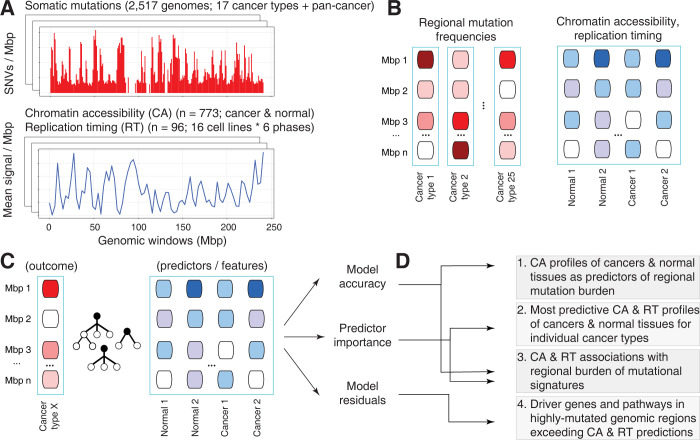
Characterising chromatin accessibility (CA) and replication timing (RT) as predictors of regional mutagenesis in cancer genomes. **A.** Somatic mutations in cancer genomes (top) and CA and RT datasets of normal tissues and cancers (bottom) were integrated to study regional mutational processes. Somatic single nucleotide variants (SNVs) of 2,517 whole cancer genomes were analysed with 869 genome-wide epigenetic profiles, including 773 CA profiles of primary human cancers, normal tissues, and cell lines from ATAC-seq experiments, and 96 RT profiles of 16 cell lines and six cell cycle phases from RepliSeq experiments. **B.** Genomic regions of one megabase (Mbp) were analysed. Regional mutation burden was estimated as the number of SNVs per megabase region. The mean values per region were derived for CA and RT profiles. **C.** Random forest models were trained using regional mutation burden profiles as the outcome and CA and RT profiles as the predictors. We analysed the pan-cancer dataset and 17 datasets of specific cancer types for which relevant CA and RT profiles were available. **D.** To associate regional mutation burden with CA and RT, mutational signatures, and cancer driver genes, the random forest models were evaluated in terms of accuracy, predictor importance, and model residuals.

To study the epigenomes of primary cancers in the context of mutational processes, we collected 773 ATAC-seq profiles of genome-wide CA measurements in primary human cancers, normal tissues and cell lines from ENCODE3, TCGA, and additional studies [30–37], as well as 96 RepliSeq profiles of DNA replication timing measurements in 16 cell lines and six cell cycle phases in ENCODE [38,39] (**Figs 1A and S1 and S1 Table**). As regional mutation burden, we studied 23 million SNVs in 2,465 highly-mappable genomic regions of one megabase from 2517 whole cancer genomes of 37 cancer types of the ICGC/TCGA PCAWG project [1] (**Fig 1B**). The 869 CA and RT profiles were derived as mean signal intensity values per megabase.

To map the complex non-linear associations of CA and RT profiles with regional mutagenesis, random forest regression models were trained with mutation burden profiles as outcomes and CA and RT profiles as predictors (*i*.*e*., features) (**Fig 1C**). We analysed 17 cancer types for which both genomic and relevant epigenomic profiles were available. The most informative predictors were quantified using statistical analysis and local feature prioritisation of random forests [40] (**Fig 1D**). As expected, genome-wide profiles of regional mutation burden clustered according to cancer types (**S2 Fig**).

We asked whether the CA profiles of cancers or those of normal cells and tissues showed stronger associations with the distribution of mutations in cancer genomes. We predicted regional mutation burden in pairs of random forest models with matched data splits where the predictors included either CA profiles of primary cancers or CA profiles of normal cells and tissues, respectively. RT profiles were also included as predictors in both models to estimate the relative contributions of CA.

**Fig 2:**
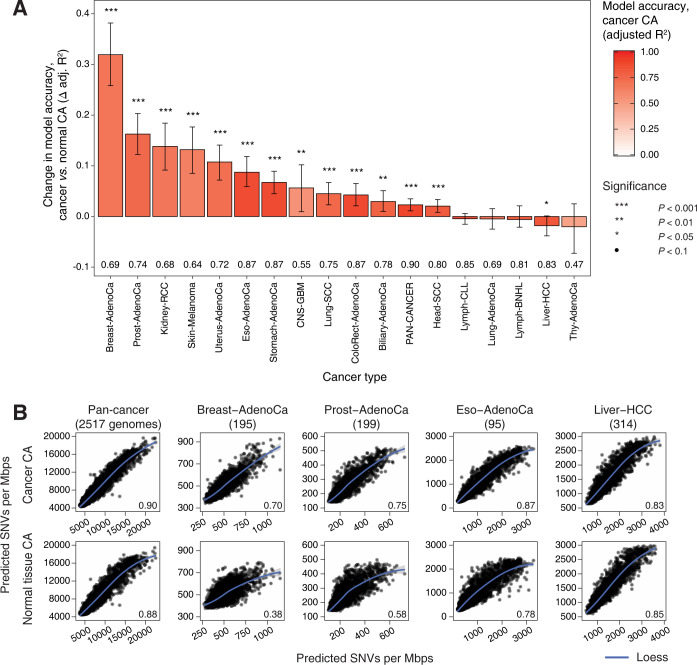
Chromatin accessibility profiles of primary cancers are stronger predictors of regional mutagenesis. **A.** Random forest models informed by CA profiles of primary cancers are more accurate predictors of regional mutation burden, compared to models informed by CA of normal tissues. Bar plot shows relative change in prediction accuracy (Δ adjusted R^2^) of random model regression models informed by CA profiles of primary cancers, compared to matching models informed by CA of normal tissues. Replication timing (RT) profiles are included in all models as reference. P-values of permutation tests and 95% confidence intervals from bootstrap analysis are shown. Accuracy values of models informed by cancer CA profiles are listed below the bars (adjusted R^2^). **B.** Examples of regional mutation burden predicted using models informed by CA profiles of cancer (top) *vs*. CA profiles of normal tissues (bottom). Scatterplots show model-predicted and observed mutation burden (X *vs*. Y-axis) in one-megabase regions. Prediction accuracy values are shown (bottom right).

In most cancer types, CA profiles of primary cancers showed stronger associations with regional mutagenesis than CA profiles of normal cells and tissues (13 of 17, *P* < 0.05) (**Fig 2A**). The most pronounced signal was observed in breast cancer where the regional mutagenesis predictions informed by cancer CA profiles were nearly twice as accurate as those informed by CA profiles of normal tissues (median adj.R^2^ 0.70 *vs*. 0.38; *P* < 0.001) (**Fig 2B**). Stronger associations of cancer CA profiles and regional mutagenesis were also found in cancers of the prostate, uterus, and kidney, and melanoma: the improvement in prediction accuracy was above 10% in those cancer types (*P* < 0.001). Stronger associations with cancer epigenomes were also confirmed in the pan-cancer analysis across 37 cancer types, with a small but statistically significant improvement in model accuracy (adj.R^2^ 0.90 *vs*. 0.88; *P* < 0.001). As the only exception, regional mutation burden in liver cancer better associated with CA profiles of normal tissues (adj.R^2^ 0.85 *vs*. 0.83; *P* = 0.044). The analysis provided inconclusive evidence for four cancer types including lymphoid cancers (BNHL, CLL) and lung and thyroid adenocarcinomas. We confirmed that the accuracy of regional mutation burden predictions in individual cancer types was not significantly correlated with the overall mutation burden or the number of sequenced genomes per cancer cohort (**S3 Fig**). To confirm that the improved accuracy of cancer CA profiles was not an effect of a larger number of predictors of this type, we performed down-sampling to provide equal numbers of epigenomes of primary cancers and related normal tissues for mutation burden predictions (**S4 Fig**).

In summary, this analysis shows that in most cancer types, regional mutagenesis is more strongly associated with chromatin accessibility of primary human cancers rather than normal tissues and cell lines, even when accounting for DNA replication timing in the comparison. The diverse collection of epigenomes included as predictors suggests that tissue-specific chromatin features of individual cancer types, as well as pan-cancer chromatin features of proliferative cells may contribute to regional mutagenesis.

### Top predictors of regional mutagenesis match cancer types and sites of origin

**Fig 3:**
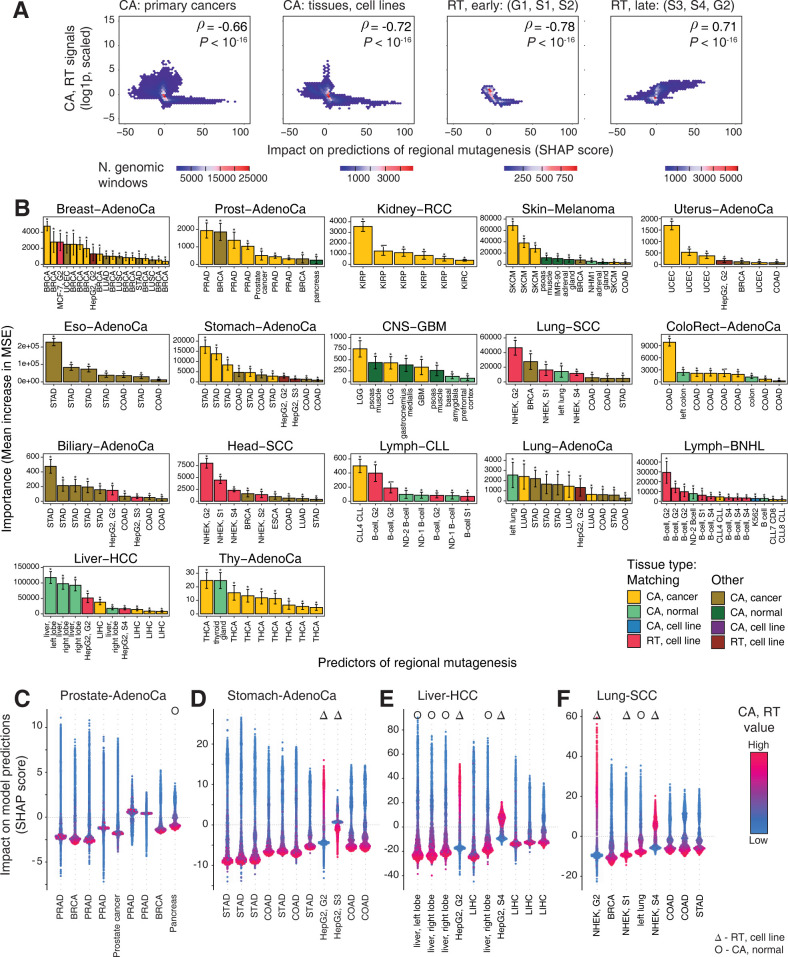
Top predictors of regional mutagenesis tie cancer types and sites of origin. **A.** 2D-density plots show the association CA and RT scores (Y-axis) and Shapley feature importance (SHAP) scores in each genomic window (X-axis) across all cancer types for significant predictors (permutation *P* < 0.001). CA profiles for cancer and normal samples, early RT profiles, and late RT profiles are plotted separately. CA and early RT profiles negatively correlate with regional mutation burden while late RT profiles correlate positively. Spearman correlation values are shown (top right). **B.** CA profiles of primary cancers are the top predictors of regional mutagenesis in most cancer types. Bar plot shows the importance scores of the significant predictors of random forest models for 17 cancer types (permutation *P* < 0.001). Error bars show ±1 standard deviation (s.d.) from bootstrap analysis. Brighter colors indicate the predictors where the epigenomic profile (CA or RT) matches the mutation profile of the related cancer type. **C-F.** Shapley additive explanation (SHAP) scores of significant predictors. SHAP scores show the impact of a given predictor on the predictions of regional mutation burden (Y-axis) relative to the values of the predictor (CA or RT; color gradient). In CA profiles, higher CA values (purple) and negative SHAP scores show decreased mutation burden in open chromatin. In late RT profiles, higher RT values and positive SHAP scores show increased mutation burden. Symbols indicate RT profiles (triangles) and CA profiles of normal tissues (circles). SHAP scores for all significant predictors are shown in **S6 Fig**. Duplicated labels of in panels B-F indicate the CA profiles of different cancer samples in the TCGA dataset (**S1 Table**).

To interpret our predictive models of regional mutagenesis, we asked which specific CA and RT profiles contributed most to the predictive models. We selected the most significant predictors for each cancer type from all 869 cancer and normal epigenomes (permutation *P* < 0.001). These 166 CA and RT profiles were quantified using Shapley additive explanation (SHAP) scores [40] that measured the directional associations of individual profiles with regional mutation burden in all genomic regions. As expected, regional mutation burden negatively correlated with CA profiles of primary cancers and normal tissues (ρ_cancer_ = -0.66 vs ρ_normal_ = -0.72; *P* < 10^−16^) (**Fig 3A**). A dual relationship was apparent in RT profiles: the RT profiles of late cell cycle phases positively correlated with regional mutation burden while the RT profiles of early phases correlated negatively (ρ_late_ = 0.71 *vs*. ρ_early_ = -0.78, *P* < 10^−16^). As controls, we reviewed the SHAP scores of non-significant predictors and expectedly found much smaller values, indicating their reduced impact on mutation burden predictions (**S5 Fig**). The inverse relationships of CA and RT with respect to regional mutation burden are consistent with previous studies [17–23] and extend here to a diverse collection of epigenomes from primary cancers and normal tissues.

We examined the most significant predictors of regional mutation burden for each cancer type (*P* < 0.001) (**Figs 3B and S6**). CA profiles of primary cancers were enriched among the top predictors (111 of 166 profiles observed *vs*. 80 expected, Fisher’s exact *P* = 8.7 x 10^−8^), confirming the stronger association with cancer epigenomes and regional mutagenesis. Furthermore, most CA profiles represented the same or related cancer type in which the regional mutation burden was predicted. Regional mutation burden measured in the breast, prostate, kidney, stomach, and thyroid cancer genomes of the PCAWG project associated with the CA profiles of the matching cancer samples of the TCGA project (BRCA, PRAD, KIRP, STAD, and THCA, respectively). For example, nine significant predictors were found in prostate cancer, including six CA profiles of prostate cancers, two CA profiles of breast cancer, and one CA profile of normal pancreas (**Fig 3C**). Additional associations were apparent at the level of organ systems. CA profiles of stomach and colorectal cancers were the top predictors of regional mutation burden in stomach, biliary and esophageal cancers (**Fig 3D**), suggesting epigenetic and mutational similarities of gastrointestinal cancers. Therefore, regional mutational processes in individual cancer types have strong tissue-specific interactions with the epigenomes of these cancer types.

Fewer CA profiles of normal tissues were found among top predictors of regional mutation burden. The strongest association with normal tissue epigenomes was apparent in liver cancer, as three CA profiles of normal liver were detected as the highest-ranking predictors of regional mutation burden, followed by CA profiles of liver cancers and RT profiles of the liver cell line HepG2 (**Fig 3E**). CA profiles of normal tissues were found as predictors in several other cancer types; however, their feature importance scores were substantially lower compared to the CA profiles of related primary cancers. For example, in thyroid cancer, only one normal thyroid CA profile was found as a significant predictor of regional mutagenesis, in addition to eight primary cancer CA profiles of the matching cancer type (THCA). As expected, CA profiles of normal tissues also matched the cancer types where regional mutagenesis was measured.

Replication timing showed the strongest associations in squamous cell cancers (SCC) and lymphoid cancers, reflecting tissue-specific effects. Mutations in Lung-SCC and Head-SCC associated with RT profiles of NHEK cells, a squamous cell line of human epidermal keratinocytes (**Fig 3F**). Similarly, regional mutation burden in lymphoid cancers (Lymph-BNHL, Lymph-CLL) strongly associated with RT profiles of B-cells. An earlier occurrence of mutagenesis in the evolution of these cancer types is one potential explanation of the associations with these normal cell lines. In squamous cell cancers of lung and head & neck, many mutations are associated with tobacco exposure signatures, while somatic hypermutation found in lymphomas also contributes to genome variation in normal B-cells [41].

Most RT predictors of regional mutagenesis represented late-replicating cell cycle phases G2 and S4. Individual RT profiles positively associated with regional mutation burden in late-replicating regions (*e*.*g*., phase G2 of MCF-7 in breast cancer) and negatively in early-replicating regions (*e*.*g*., phase S1 of HNEK in Head-SCC) (**Fig 3A**). This is consistent with our analysis above and with earlier observations that elevated regional mutagenesis is caused by increased DNA damage and decreased repair in late-replicating regions [20].

Overall, we found fewer significant RT profiles compared to CA profiles. However, these also matched the expected cells of cancer origin: for example, the RT profiles of related cell lines (MCF-7, HepG2) were found among predictors of regional mutation burden in breast and liver cancer, respectively. Fewer and less-diverse RT profiles of cell lines were available for this analysis, and these provide only a limited representation of mutational processes in different cancer types. In contrast, the larger set of CA profiles represents more cancer types and provides complementary information to RT. This analysis extends our findings of tissue-specific CA and RT profiles as the principal predictors of regional mutagenesis in cancer. It also underlines the effects of cell-of-origin and tumor heterogeneity. Dominance of cancer CA profiles among top predictors in most cancer types is consistent with our first observations that CA profiles of primary cancers provide more accurate predictions of regional mutagenesis.

### Associations of mutational signatures with chromatin accessibility and replication timing

We asked whether the associations of regional mutagenesis with CA and RT were further explained by mutational signatures. We quantified the megabase-scale mutation burden separately for the major single base substitution (SBS) signatures based on PCAWG datasets [6] and predicted their regional distributions using random forest regression. We then selected the CA and RT profiles that significantly associated with each mutational signature (*P* < 0.001).

**Fig 4:**
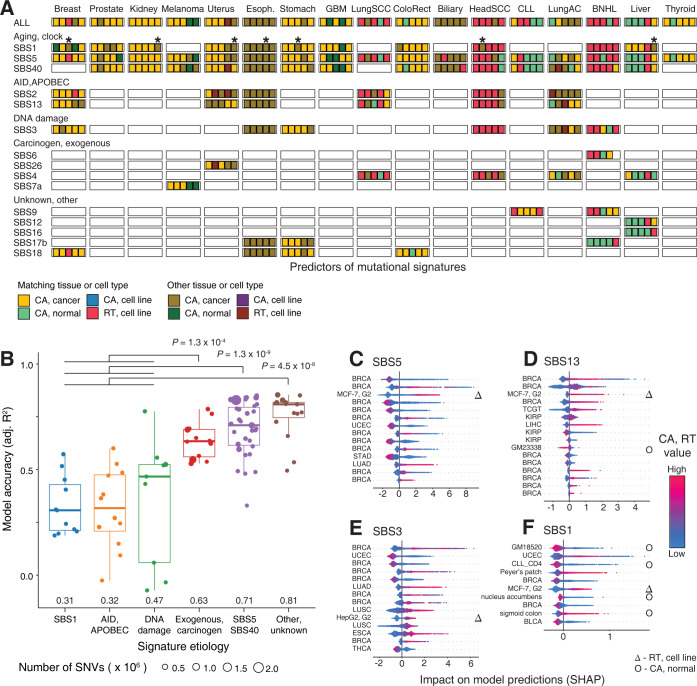
Associations of mutational signatures with chromatin accessibility and replication timing. **A.** Top predictors of megabase-scale mutation burden of single base substitution (SBS) signatures (top five predictors; *P* < 0.001, permutation test). Colors indicate the predictor type (CA, RT) and its relationship to the cancer type where mutagenesis is predicted (matching site/tissue or other). Brighter colors indicate the predictors where the epigenomic profile (CA or RT) matches the cancer type of the mutation profile. Asterisks indicate CA profiles of CD4-positive T-cells as predictors of SBS1 mutations. All significant predictors are shown in **S7B Fig.** Prediction accuracy of megabase-scale burden of SBS signatures using CA and RT profiles. Signatures of carcinogens, unknown origin, and aging are more accurately predicted by CA and RT profiles than endogenous signatures. *P*-values are computed using F-tests with adjustment for genome-wide signature burden. Median accuracy values are printed. **C-F**. SHAP scores of significant predictors of selected mutational signatures in breast cancer. SHAP scores show the impact of the predictor (*i*.*e*., CA or RT profile) on the predictions of SBS burden (X-axis) and the corresponding predictor values (color gradient). Symbols indicate RT profiles (triangles) and CA profiles of normal tissues (circles). SHAP scores for all significant SBS predictors for breast cancer are shown in **S8 Fig**.

We compared the significant CA and RT profiles that associated with individual mutational SBS signatures (**Figs 4A and S7**). Top predictors of individual signatures showed tissue-specific associations of mutagenesis and chromatin accessibility, similarly to overall mutational profiles. CA profiles of matching cancer types were the top predictors of mutational signatures in breast, kidney, colorectal and stomach cancers, while the RT profiles of matching cell lines associated with mutations in SCCs and lymphoid cancers. These significant predictors of SBS mutations were often consistent with the predictors of overall SNV burden. The top predictors of endogenous and exogeneous signatures were also mostly consistent with those of overall mutation burden, indicating that various mutational processes are often affected by the epigenetic landscapes of cancer cells.

SBS1 mutations associated with the most diverse set of CA profiles across cancer types. Interestingly, the CA profile of CD4-positive T-cells from the peripheral blood of a CLL patient (CLL1_CD4) was consistently detected as a top-ranking predictor of SBS1 mutation burden in six solid cancer types (liver, kidney, uterus, esophagus, stomach, head). This CA profile was only specific to SBS1 mutations and was not associated with bulk mutation burden or any other SBS signatures in the cancer types we studied. This T-cell CA profile may represent somatic mutations in non-cancerous cells of the immune system or the tumor microenvironment. As another example, SBS1 mutations in liver cancer associated with CA profiles of liver cancers, in contrast to the overall mutational profile that was predominantly associated with the CA profiles of normal liver tissues. The clock-like SBS1 signature of 5-methylcytosine deamination is associated with cancer patient age and stem cell division rate, and this signature has been found in the somatic genomes of normal tissues and adult stem cells [10,42,43]. Thus, SBS1 mutations may represent an earlier timepoint in tumor evolution or the somatic genome variation of normal cells.

We asked if our random forest models were equally informative of various mutational signatures. Six classes of SBS signatures were compared in terms of prediction accuracy: APOBEC/AID, DNA-repair, carcinogens, two age-related classes (SBS1 and SBS5/40), and signatures of unknown cause, as predicted in 17 cancer types through the dataset of 869 CA and RT profiles. Three classes of signatures showed stronger associations with CA and RT profiles: random forest predictions of carcinogenic signatures, signatures of unknown cause, and aging-associated signatures SBS5 and SBS40 were significantly more accurate than the predictions of SBS mutations associated with DNA repair, APOBEC/AID, and SBS1 signatures (**Figs 4B and S9**). For a more conservative estimate, this analysis accounted for the number of mutations per signature as covariate of prediction accuracy (*P* ≤ 10^−4^; F-test). Thus, the mutational processes of carcinogen exposures, SBS5/40, and unannotated signatures show stronger interactions with CA and RT in cancer genomes.

We studied the interactions of different SBS signatures with CA and RT profiles in breast cancer (**Figs 4C–4F and S8**). SBS5 was mostly predicted by CA profiles of breast cancers (10 of 14 significant predictors) and CA showed mostly negative associations with mutation burden (**Fig 4C**). In contrast, SBS13 and SBS2 mutations corresponding to AID/APOBEC processes, as well as SBS3 mutations corresponding to DNA repair, mostly correlated positively with CA, such that higher SHAP values and higher mutation load corresponded to increased chromatin accessibility (**Fig 4D–4E**). In agreement with this, prior observations have shown that AID targets epigenetically active elements and results in kataegis and clustered mutational signatures [6,44,45]. Lastly, SBS1 mutations associated with additional CA profiles of normal cells: peripheral blood, lymphoid follicles (Peyer’s patch), and immune cells (**Fig 4F**), perhaps reflecting somatic mutagenesis in tumor-infiltrated immune cells and other cells in the tumor microenvironment. The positive and negative interactions of CA and RT with regional mutagenesis in certain mutational signatures may reflect inter- and intra-tumoral heterogeneity and help characterise these mutational processes mechanistically.

### Genomic regions with overrepresented mutations converge to cancer genes and developmental pathways

To quantify the regional mutation burden that remained unexplained by CA and RT, we investigated the genomic regions that were overrepresented in mutations relative to the mutation burden predicted by random forest regression. To enable a more detailed, gene-level functional analysis of the overrepresented mutations, we repeated the predictions of regional mutation burden using a finer resolution of 100-kbps genomic regions. We confirmed that cancer CA profiles were the major predictors of mutagenesis across 100-kbps regions, however SBS predictions were significantly less accurate due to sparser data at this increased genomic resolution (**S10 Fig**).

**Fig 5:**
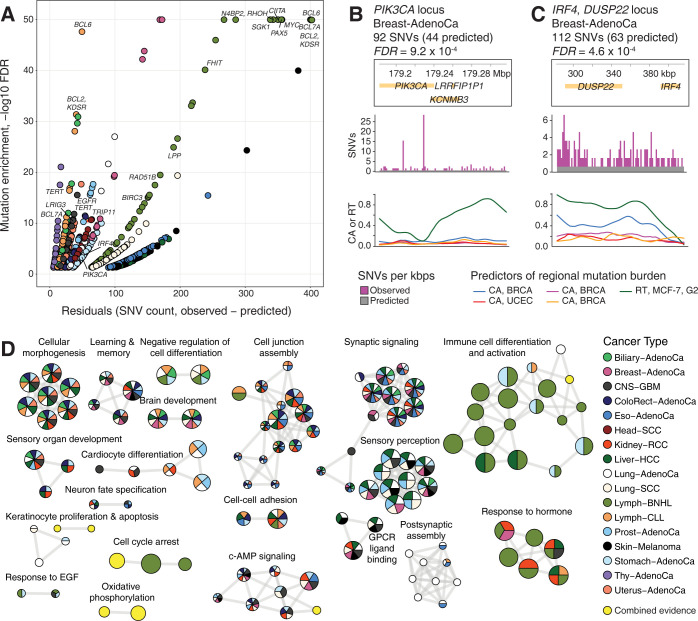
Genomic regions with overrepresented mutations converge to cancer genes and developmental pathways. **A.** Genomic regions (100-kbps) with overrepresented mutations that significantly exceeded the predictions of models informed by CA and RT profiles. Regions with significantly higher model residuals are shown (*FDR* < 0.05). Significant regions with known cancer genes are labeled (*FDR* < 10^−10^). Colors indicate the cancer types where the overrepresented mutations were found (see panel D). X-axis is capped at 50 and Y-axis is capped at 400. **B-C**. Examples of genomic regions with overrepresented mutations and known or putative cancer driver mutations. The plots show the genes in the region (top), observed and expected mutation burden (SNVs per kbps; middle), and the top five most significant predictors in our models (*i*.*e*., CA and RT profiles; bottom). **B.** The genomic region encoding the driver gene *PIK3CA* in breast cancer. **C.** Super-enhancer region at the *IRF4*-*DUSP22* locus with overrepresented mutations in breast cancer. **D.** Pathway enrichment analysis of the genomic regions where the mutations were overrepresented compared to CA- and RT-informed predictions. The enrichment map visualisation shows significantly enriched biological processes and pathways (*FDR* < 0.05). Nodes in the network represent enriched pathways and processes, the edges connect nodes sharing many genes, and the manually annotated sub-networks represent functionally related pathways and processes. Colors show the cancer types where the pathway enrichments were detected. The pathways and processes that were only detected in the joint analysis of multiple cancer types are shown in yellow.

We then selected specific genomic regions where the models informed by CA and RT profiles strongly underestimated the observed mutation burden in individual cancer types. This revealed 1570 genomic regions in 17 cancer types that were significantly overrepresented in mutations based on the CA- and RT-informed model residuals (*FDR* < 0.05) (**Fig 5A**). The mutation-enriched regions encoded 900 protein-coding genes including 67 known cancer genes [46], significantly more than expected by chance (33 expected, Fisher’s exact *P* = 3.1 x 10^−8^). Most driver genes were only found in single cancer types and represented key disease-specific drivers such as *EGFR* and *TERT* in glioma, *MYC* in BNHL, and *APC* in colorectal cancer (**S11 Fig**). For example, in breast cancer, the region encoding *PIK3CA* was significantly overrepresented in mutations, compared to the expected mutation burden predicted from the models based on CA and RT profiles (92 SNVs observed *vs*. 44 expected; FDR = 9.2 x 10^−4^) (**Fig 5B**). *PIK3CA* is a major driver gene of breast cancer with well-defined hotspot mutations [47], thus showing that our genome-wide machine learning models of CA and RT can capture known driver genes.

The models also revealed novel regions with frequent non-coding mutations. The most prominent region was found due to unexpectedly frequent mutations that were found in 12 of 17 cancer types including breast cancer (112 SNVs observed *vs*. 63 expected; FDR = 4.6 x 10^−4^) (**Figs 5C and S12**). The region includes the oncogenic transcriptional regulator *IRF4* (interferon regulatory factor 4) [48], as well as the *DUSP22* gene encoding a signaling protein that was recently described as a network-implicated driver gene due to non-coding mutations [49]. The region also includes super-enhancers of immune cells [50]. The recurrence of mutations in this region in multiple cancer types highlights it as a potential pan-cancer region of interest.

We then asked whether the frequently mutated regions were associated with common biological pathways and processes. We revealed 177 significantly enriched pathways and processes using an integrative pathway enrichment analysis method that prioritised genomic regions detected in multiple cancer types (*FDR* < 0.05, ActivePathways [51]) (**Fig 5D**). Of those, 142 (80%) were detected in more than one cancer type. These findings converged into several functional themes. First, developmental processes related to brain and the central nervous system, reproductive, and sensory organs were associated with elevated mutation burden in multiple cancer types. Second, a group of processes related to synapse organisation, olfactory and GPCR signaling were also identified due to frequent mutations in most cancer types. Third, cancer-related processes of cell cycle, hormone response, and signal transduction were also identified, often through pan-cancer data integration but not in any specific cancer type specifically. Lastly, a major group of processes related to immune system activation were predominantly detected in BNHL, as well as in liver and stomach cancers to a smaller extent.

This analysis shows that although individual frequently mutated genomic regions are mostly characteristic of specific cancer types, overrepresented mutations converge to common pathways and processes in multiple cancer types. Convergence of these overrepresented mutations to developmental and cancer-related processes is potentially explained by additional focal mutational processes targeting epigenetically active regions of the genome that are not captured by our models at the broader, sub-megabase resolution. Further, the enrichment of known cancer driver genes suggests that positive selection of functional mutations may also contribute to this overrepresentation of mutations. This analysis exemplifies the complex interplay of cancer epigenomes, multi-scale mutational processes and positive selection of cancer genes.

## Discussion

Our analysis shows that chromatin accessibility of primary human cancers provides the strongest associations with regional mutational processes in cancer genomes. Cancer epigenomes are predictive of regional mutation burden of matching cancer types, indicating the tissues of origin in most cancer types we studied. While these associations are apparent for overall regional mutation burden in cancer genomes, the signals are also consistent for various mutational signatures. In contrast, the epigenomes of normal tissues and cell lines show only limited associations with regional mutagenesis of cancer genomes, complementing the earlier studies that used the epigenetic profiles of cell lines and normal tissues to characterise mutational processes. The transformation of normal cells to cancer cells involves major epigenetic changes as gene-regulatory programs of cancer hallmark pathways are activated. Thus, one potential explanation to this stronger association of cancer epigenomes and regional mutagenesis is that mutational processes are more active in rapidly dividing cancer cells. Thus, many passenger mutations occur later in cancer evolution after the cells have acquired the epigenetic characteristics of cancer cells.

DNA replication timing also associated with regional mutagenesis and highlighted the cell types of cancer origin. However, CA profiles of primary human cancers were better predictors of regional mutagenesis compared to RT profiles, apart from squamous cell and lymphoid cancers that strongly associated with the RT profiles of relevant cell lines. Fewer RT profiles were used as predictors in our models, and these included mitotic cell lines that offer only limited representation of the diverse pan-cancer dataset. As our models also include RT profiles of several cell lines as reference, the stronger association with the CA profiles of primary cancers shows that cancer epigenomes complement RT information with respect to regional mutagenesis. Interestingly, DNA replication has been shown to determine chromatin state [52]. However, comparison of prostate cancer cells and normal cells suggested that DNA replication timing is largely conserved between the two states [25]. Thus, the CA profiles of human cancers that we found as stronger predictors of regional mutagenesis may jointly represent chromatin-specific as well as replication-specific contributions to mutational processes.

Mutational signature analysis revealed numerous interactions of mutational processes with cancer epigenomes and tissues of origin. Carcinogen-induced signatures, the aging-associated signatures SBS5/40, as well as signatures of unknown etiology, were overall better predicted by CA and RT, in contrast to the endogenous signatures of AID/APOBEC and DNA repair processes for which the genome-wide predictions were less accurate. The stronger association of carcinogen signatures with CA and RT profiles suggests that the chromatin environment interacts with DNA damage or repair processes of carcinogen exposure, for example through elevated mutational processes targeting active genes that are otherwise protected from mutations through error-free mismatch repair [45]. Similarly, early-replicating regions in cells exposed to tobacco mutagens show elevated mutagenesis in transcribed strands due to differential nucleotide excision repair activity [53]. Analysis of mutational processes in the three-dimensional genome shows that carcinogenic signatures are dominant in inactive topologically associating domains while active domains are more characterised by endogenous signatures [29]. Based on their stronger interactions with RT and CA profiles, we speculate that some mutational signatures of currently unknown etiology may relate to carcinogens. For example, SBS17 mutations show some of the strongest interactions with CA and RT in stomach and esophageal cancers in our analysis. This signature is currently of unknown cause, however it has been linked to gastric acid reflux and reactive oxygen species [24]. Further integrative analysis of clinical and lifestyle information with patterns of regional mutagenesis may shed light to these mutational processes.

We observed a functional convergence to developmental processes and cancer-related pathways in the genomic regions where mutations were overrepresented beyond the predictions of our epigenomic models. On the one hand, these data suggest that some additional mutational processes affect open-chromatin regions encoding developmental genes in individual cancer types. However, these regions converge to the same molecular pathways across cancer types. Such local mutational processes are consistent with previous studies. For example, transcription start sites of highly expressed genes and constitutively-bound binding sites of CTCF are subject to elevated local mutagenesis in multiple cancer types [16]. Lineage-specific genes are enriched in indel mutations in solid cancers [54]. Such local mutational processes are complementary to megabase-scale processes in which open chromatin is generally associated with a lower mutation frequency. On the other hand, the enrichment of cancer genes and pathways in our data suggests that some mutations unexplained by CA and RT profiles are functional in cancer, and their frequent occurrence at specific genes, non-coding elements and molecular pathways is explained by positive selection [3–5, 49]. We can use this computational framework to find genomic regions with known and putative driver mutations in coding and non-coding sites. Further study of these regions may deepen our understanding of mutational processes and refine the catalogues of driver mutations.

We used the random forest method to study the regional characteristics of mutational processes in cancer genomes. The approach has several benefits for this purpose: the models can detect non-linear relationships, are relatively transparent, and work well in the small datasets provided by the megabase-scale genomic resolution. Interpreting the models systematically using permutations, bootstrap analysis, and SHAP scores allowed us to learn the tissue-specific interactions of regional mutational processes, SBS signatures, chromatin states and DNA replication, while our analysis of model residuals provided further insights into driver mutations and local mutagenesis. However, the way we included non-overlapping genomic regions discards their higher-order interactions. These may be captured better with alternative methods such as hidden Markov models or recurrent neural networks that consider adjacent genomic regions sequentially.

Our approach enables future studies to decipher the mechanisms and phenotypic associations of mutational processes. Clinical, genetic, and epigenetic profiles of cancer patients can be integrated to understand how regional mutational processes and the chromatin landscape are modulated by clinical variables such as stage, grade or the therapies applied, genetic features such as somatic driver mutations or inherited cancer risk variants, and lifestyle choices such as tobacco or alcohol consumption. Complementary insights from sub-clonal reconstruction analysis of cancer genomes [2,55], as well as single-cell sequencing of genomes and epigenomes will allow mapping of regional mutagenesis at the level of distinct cell populations contributing to temporal and spatial variation in mutational processes. As such multimodal datasets grow, we can learn about early cancer evolution by comparing regional mutagenesis in the genomes of cancers and normal cells. Understanding the molecular and genetic determinants of regional mutagenesis and signatures in cancer genomes may help characterise carcinogen exposures and genetic predisposition, ultimately enabling and enhancing early cancer detection and prevention approaches in the future.

## Methods

### Human subjects

Previously published genomics datasets were used for our analysis. DNA sequencing of human subjects’ tissue was performed by ICGC and TCGA consortium members outside of this study under a series of locally approved Institutional Review Board (IRB) protocols as described earlier [1]. Informed consent was obtained from all human participants as part of previous studies. Ethical review of the current data analysis project was granted by the University of Toronto Research Ethics Board (REB) under protocol #37521.

### Somatic mutations in whole cancer genomes

We analysed somatic single nucleotide variants (SNVs; n = 43,778,859) derived from whole-genome sequencing (WGS) of 2,583 primary cancer samples that were uniformly mapped to GRCh37/hg19 as part of the ICGC/TCGA Pan-cancer Analysis of Whole Genomes (PCAWG) project [1]. Indels and variants in sex chromosomes were excluded. To integrate mutations with epigenetic information, we mapped the SNVs to the human genome version GRCh38 using the LiftOver function of the rtracklayer package in R (v 1.48) [56]. We removed 66 hypermutated tumors with more than 90,000 mutations (~30 mutations / Mbps), resulting in a dataset of 23,215,600 SNVs in 2,517 whole cancer genomes. We analysed the genomes of 17 cancer types with at least 25 samples in PCAWG as well as related epigenetic profiles of normal and cancer tissues, as well as the pan-cancer dataset of all 37 cancer types.

### Chromatin accessibility (CA) and replication timing (RT) profiles

Chromatin accessibility data was derived from several ATAC-seq datasets, including the ENCODE3 project and six additional studies to maximise the coverage of cancer types included in the PCAWG dataset, as described below. CA profiles of 196 human cell and tissue types and 9 cancer cell lines at a single basepair (bp) resolution in GRCh38 were derived from the ENCODE3 project [30]. CA profiles of 115 normal human brain samples at a single bp resolution in GRCh37 were retrieved from the study by Fullard *et al*. [32]. CA profiles of 21 normal immune cell types (B-cells, T-cells) and 34 primary cancers (CLL) at a single base pair resolution in GRCh37 were retrieved from the study by Rendeiro *et al*. [33]. Four CA profiles of HEK293 embryonic kidney cells at a 10-bp resolution in GRCh37 were retrieved from the study by Karabacak Calviello *et al*. [34]. CA profiles for two lymphoma cell lines at a single-bp resolution in GRCh37 were retrieved from the study by Scharer *et al*. [35]. One CA profile of the normal human melanocytes (NHM1) cell line at a 10-bp resolution in GRCh37 were retrieved from the study by Fontanals-Cirera *et al*. [36]. CA profiles of four normal prostate tissues and six primary prostate cancers at a single-bp resolution in GRCh37 were retrieved from the study by Pomerantz *et al*. [37]. CA profiles of several cancer types were retrieved from the TCGA ATAC-seq dataset [31] of 410 primary cancer samples, representing cancers of 404 unique patient donors and 796 genome-wide profiles in total. We used 381 CA profiles of the TCGA dataset such that technical and biological replicates of distinct cancer samples were pooled by per-region averaging of CA signal. Prior to this averaging, 22 CA profiles with only one replicate available were removed, and one CA profile of a low-grade glioma (LGG) that was an outlier in our initial analyses was also removed. In total, 773 CA profiles were included in the analysis, including 421 cancer profiles, 341 profiles of normal tissues and cell lines, and 11 profiles of cancer cell lines. Besides the CA profiles, 96 replication timing profiles of 16 cell lines, each with six cell cycle phases, were derived from the RepliSeq study (GRCh37) in the ENCODE project (GEO accession GSE34399) [38,39]. CA and RT profiles were constructed from the BigWig files of the original studies using mean values of signal intensity per each genomic window. Genomic coordinates of the GRCh37 reference genome were mapped to the GRCh38 reference genome using LiftOver. In total, the set of 869 (773 + 96) CA and RT profiles was used.

### Integrating regional mutagenesis with CA and RT profiles

We evaluated chromatin accessibility, replication timing and mutation burden in non-overlapping genomic regions of one megabase (Mbp; one million base pairs). We excluded a subset of genomic regions with low mappability (≤80% in the UMAP software [57]) as well as sex chromosomes, resulting in 2,465 regions included in the study. For megabase-scale CA and RT profiles, each genomic region was assigned the mean value of its epigenetic signal. For megabase-scale somatic mutation burden, each region was assigned the total mutation count separately for the pan-cancer dataset and each of the 17 cancer types. In two cohorts (chronic lymphocytic leukemia; B-cell non-Hodgkin lymphoma), we removed two regions encoding immunoglobulin genes (chr2, 89 Mbps; chr22, 23 Mbps) with known high somatic variation in immune cells, as observed in our initial analyses.

### Random forest regression

Megabase-scale profiles of mutation burden and CA and RT profiles were analysed with random forest regression [58] with CA and RT profiles as the predictors (*i*.*e*., features) and mutation burden as the target (*i*.*e*., response). Number of trees (1000) and fraction of predictors at each split (1/3) were used as hyperparameters. Monte-Carlo cross-validation over 1000 data splits considered subsets of genomic regions for model training (80%) and validation (20%). We used the adjusted R^2^ (adj.R^2^) metric to evaluate model performance that measures the variance explained by the model adjusted for model complexity (*i*.*e*., the number of CA and RT profiles used for predictions).

### Comparing CA profiles of primary cancers and normal tissues as predictors of regional mutagenesis

First, we compared the overall accuracy of predicting megabase-scale mutation burden using CA profiles of cancers *vs*. normal tissues. Two sets of random forest regression models were run in a joint Monte-Carlo cross-validation procedure that used all CA profiles of normal tissues (M_n_) and cancers (M_c_) as predictors, respectively. Both models also included the same set of RT profiles as predictors as reference. At each iteration, models were trained on matching subsets of genomic regions (80%) and tested on the remaining genomic regions (20%), and model accuracy (adj.R^2^) values as well as the relative change values (Δadj.R^2^ = adj.R^2^(M_c_)- adj.R^2^(M_n_)) were derived in the corresponding test sets, allowing us to directly compare the two models. For each cancer type, median Δadj.R^2^ values and 95% confidence intervals were reported. Empirical P-values were computed as the fraction of cross-validation iterations where the Δadj.R^2^ crossed on the opposite side of zero relative to the median value. We also trained the models M_n_ and M_c_ on the full set of genomic regions and compared the accuracy of the two sets of models. Observed and model-predicted mutation burden values per region were visualised as scatter plots with local regression (loess) trendlines (span = 0.9). Spearman correlation tests were used to evaluate the associations of model accuracy, WGS cohort size and per-megabase mutation burden in different cancer types. We repeated most analyses using 100-kbps genomic windows and confirmed that CA profiles of primary cancers provided improved accuracy compared to non-cancer CA profiles. Non-parametric analysis with paired Wilcoxon rank-sum tests was used to compare model accuracy at the 1-Mbps and 100-kbps resolutions. We also down-sampled predictors by randomly sampling subsets of predictors for individual cancer types for 1000 iterations such that the random forest models included equal numbers of CA profiles of normal tissues and cancer tissues. This down-sampling confirmed that the observed improvement in model performance with cancer CA profiles was not caused by biases in predictor composition.

### Evaluating CA and RT profiles as predictors of regional mutation burden

We used the metric of incMSE (increase in model mean-squared-error) to evaluate the most important features (*i*.*e*., CA, RT profiles) in the random forest models. incMSE measures the relative change in model prediction accuracy upon permutations of the values of a given feature. We derived incMSE values of CA and RT profiles for the 17 cancer types for which matching CA and/or RT profiles were available. Two additional statistical methods were used to evaluate the significance of incMSE of CA and RT profiles. First, permutation tests were used to detect CA and RT profiles where incMSE values significantly exceeded those observed in permuted data. We fitted random forest regression models for every cancer type 1,000 times using randomly reassigned megabase-scale mutation burden estimates as null distributions for incMSE values for CA and RT profiles. Specific profiles were considered statistically significant if their observed incMSE values exceeded all 1000 incMSE values from permuted datasets (*i*.*e*., empirical *P* < 0.001). Second, we used bootstrapping of random forest regression where the genomic regions with predictor and response values were sampled randomly with replacement. We repeated the resampling 1000 times and recorded the incMSE values for all CA and RT profiles to evaluate the confidence intervals of the derived incMSE values.

### Feature importance of CA and RT profiles in predictions of regional mutagenesis

We used the Shapley Additive explanation (SHAP) method [40,59] to interpret the interactions of regional mutation burden and CA and RT profiles. Here, SHAP scores reflect the importance of each feature in the random forest model (*i*.*e*., CA or RT profile) in predicting a specific observation (*i*.*e*., mutation burden of a certain genomic region), and represent its relative contribution to the prediction (*i*.*e*., effect size) as well as the direction of the prediction (*i*.*e*., positive or negative). SHAP values were computed using models trained all genomic regions and separately for cancer types, using the python packages shap (0.35.0) [40,59] and scikit-learn (0.23.1) [60] via the R package reticulate (1.16) [61]. Two-dimensional density plots of SHAP values and corresponding CA and RT profiles were plotted for all significant predictors in various cancer types (permutation *P* < 0.001) and as controls for the non-significant predictors (*P* > 0.2). The non-significant predictors were additionally down-sampled to match the number of significant predictors in each cancer type.

### Associating mutational signatures with CA and RT profiles

For the analysis of mutational signatures, we used the catalogue of single base substitution (SBS) signatures of SNVs derived in the PCAWG project [6]. For each genomic region, we computed the mutational signature burden probabilistically by adding the SBS-specific probabilities of all individual SNVs in the region. This corresponded to the mean values of SNVs of various SBS signatures from the underlying multinomial distributions. Thus, for each SNV, the approach accounted for all potential signature exposures rather than only the top-ranking signature. We filtered lower-frequency SBS signatures in each cancer type (*i*.*e*., <20,000 SNVs). We confirmed that these probabilistic estimates of mutation burden were highly correlated with an alternative approach of selecting the top-ranking SBS for each trinucleotide-annotated SNV (**S13 Fig**). To evaluate CA and RT profiles as predictors of megabase-scale mutational signature burden, we trained random forest models where a probabilistic SBS profile was used as model response. We evaluated model performance, selected top features, and computed SHAP scores similarly to the analysis of bulk mutation analysis described above. This analysis was also repeated for the 100-kbps genomic resolution. We grouped the mutational signatures based on their etiology according to the COSMIC database (version 3.2, downloaded in March 2021): AID/APOBEC, deficient DNA repair, exogeneous/carcinogen, unknown/other, SBS5/40 and SBS1. We used ANOVA analysis and F-tests to compare the model accuracy values for regional mutational signature burden predictions at the 1-Mbps resolution for the six classes of signatures. In the ANOVA analysis, we used the covariate of the average megabase-scale SBS burden per cancer type to account for the potential of improved predictions in cancer types with higher overall mutation burden.

### Prioritising highly mutated genomic regions exceeding CA and RT predictions

To study regional mutation burden unexplained by CA and RT profiles, we prioritised the genomic regions where the random forest predictions significantly underestimated the observed mutation burden. Random forest regression was repeated on 100-kbps regions to improve gene-level interpretation. To score the genomic regions, we subtracted the predicted mutation counts from the observed counts to derive residual values. Residuals were then Z-transformed and the resulting one-tailed *P*-values were adjusted for multiple testing using Benjamini-Hochberg FDR.

### Pathway enrichment analysis of regional mutation variation

To understand the functional importance of the genomic regions with overrepresented mutations that were unexplained by CA and RT profiles, we performed an integrative pathway enrichment analysis across the relevant cancer types using the ActivePathways method [51] (*FDR* < 0.05). Gene sets of biological processes of Gene Ontology and molecular pathways of Reactome were collected from the GMT files provided in the g:Profiler web server [62] (downloaded Feb 23^rd^, 2021) and were filtered using default settings of ActivePathways. In each cancer type, all protein-coding genes were assigned the P-values reflecting overrepresented mutations (*i*.*e*., the mutation burden exceeding the predictions of CA and RT informed models). The data fusion procedure in ActivePathways prioritised the genes that were frequently mutated in multiple cancer types. Enriched pathways were visualised as an enrichment map [63] and the resulting sub-networks were curated manually. We also visualised the genomic regions with overrepresented mutations as a scatter plot of residual values and -log10-transformed FDR values that were capped at 400, and 10^−50^, respectively. We highlighted known cancer driver genes of the COSMIC Cancer Gene Census database [46] (downloaded Mar 26^th^ 2021) and evaluated their enrichment in the list of pathway-associated genes using a Fisher’s exact test.

## Supporting information

S1 Table Chromatin accessibility (CA) and DNA replication timing (RT) profiles used in the study. **A.** List of genome-wide predictors used in the study. **B.** List of cancer types from TCGA used in the study.
(XLSX)

S1 Fig Summary of whole cancer genomes of the PCAWG project included in the analysis. The rightmost column indicates whether the cancer type was included separately for regional mutation burden analysis. All cancer genomes were included in the pan-cancer analysis.
(PNG)

S2 Fig Correlation heatmap of megabase-scale mutation burden in cancer types in the PCAWG datasets. Clustering suggests similarity of cancer types in similar anatomical sites and cells of origin. For example, digestive tract cancers (stomach, esophageal, colorectal) and squamous cell cancers (lung, head & neck) are clustered.
(PNG)

S3 Fig Model accuracy of megabase-scale mutation burden prediction shows limited correlations with overall mutation burden (top) and cohort size (bottom). (PNG)

S4 Fig Down-sampling of CA predictors to match the numbers of CA profiles of primary cancers and normal tissues for each cancer type. Bar plot shows the relative changes in prediction accuracy (Δ adjusted R^2^) of random forests informed by CA profiles of primary cancers, compared to matching models informed by CA of normal tissues. RT profiles are included in all models as reference. *P*-values of permutation tests and 95% confidence intervals from the bootstrap analysis are shown. Accuracy values of models informed by cancer CA profiles are listed below the bars (adjusted R^2^).
(PNG)

S5 Fig Density plots of SHAP scores and CA and RT profiles that were predictive of regional mutation burden. The top plots show the significant features across all cancer types (permutation *P* < 0.001). The bottom row shows non-significant features as controls (permutation *P* > 0.2). The non-significant features shown were sampled randomly from all non-significant features in equal numbers to significant features of individual cancer types. Spearman correlation coefficients and *P*-values are shown.
(PNG)

S6 Fig SHAP scores of significant CA and RT predictors. Significant predictors of megabase-scale SNV burden for all cancer types are shown (*P* < 0.001). Color shows CA or RT signal (blue, low; red; high) and X axis shows impact of CA/RT values on mutation rate predictions.
(PNG)

S7 Fig All significant predictors of mutational SBS signatures (permutation *P* < 0.001). The first column shows the significant predictors of all SNVs.
(PNG)

S8 Fig SHAP scores of significant CA and RT predictors of SBS burden in breast cancer. Significant predictors of megabase-scale burden of SNVs of different SBS signatures are shown (*P* < 0.001). Colors show CA or RT signal (blue, low; red; high) and the X-axis shows impact of CA/RT values on mutation rate predictions.
(PNG)

S9 Fig Prediction accuracy of regional mutation burden of SNVs of single base substitution (SBS) signatures. (PNG)

S10 Fig Comparison of 1-Mbp and 100-kbp genomic resolution for mutation burden predictions. **A.** Improved prediction accuracy of cancer CA profiles is also observed at the finer resolution of 100-kbps genomic windows (see Fig 2A). **B.** Comparison of prediction accuracy of overall mutation burden (all SNVs, left) and SBS signatures (right). Paired Wilcoxon rank-sum P-values are shown. SBS predictions are significantly less accurate at the 100-kbps resolution. Examples of the least accurate mutation classes include the small thyroid cancer cohort that has a relatively low mutation burden, and infrequent SBS signatures for which many genomic windows have zero mutations. **C.** Heatmaps show the fractions of genomic windows with no mutations. Overall mutation burden (all SNVs) has few or no genomic windows with no mutations (top row), while the regional mutation burden of SBS signatures is less detectable at 100-kbps, and is seen in more windows with no mutations, especially in smaller cohorts and less-common SBS signatures. Data sparsity potentially explains the reduced prediction accuracy at the 100-kbps resolution.
(PNG)

S11 Fig Known cancer genes identified in the 100-kbps genomic regions where the observed mutation frequency significantly exceeds random forest predictions informed by CA and RT profiles. (PNG)

S12 Fig Regional mutation burden of the frequently mutated genomic region encoding *IRF4* and *DUSP22* (red) and the 20 adjacent regions (black). Bars represent 100-kbps regions. The predicted mutation burden from random forest models is shown in blue.
(PNG)

S13 Fig Comparison of megabase-scale mutational SBS signature burden derived using expected values and the most probable signatures. Scatter plots show the mutation counts of SBS signatures in cohorts of individual cancer types. The X-axis of each plot shows the SNVs assigned to the top-ranking signatures in each patient per 1-Mbp windows. The Y-axis shows the probabilistic assignment of all SNVs to all SBS signatures based on the expected values across the multinomial distributions of mutations in individual cancer genomes (as used in the study). Spearman correlation coefficients are shown in top-left corners of the scatter plots (all *P* < 10^−16^). Only sufficiently frequent SBS signatures were included (>20,000 SNVs per cohort based on both SBS annotation strategies).
(PNG)
