# Supplementary figures and images for: Chromatin accessibility of primary human cancers ties regional mutational processes and signatures with tissues of origin
Source: PLoS Comput Biol. 2022 Aug 10;18(8):e1010393. doi: 10.1371/journal.pcbi.1010393 (PMC9365152; doi:10.1371/journal.pcbi.1010393)

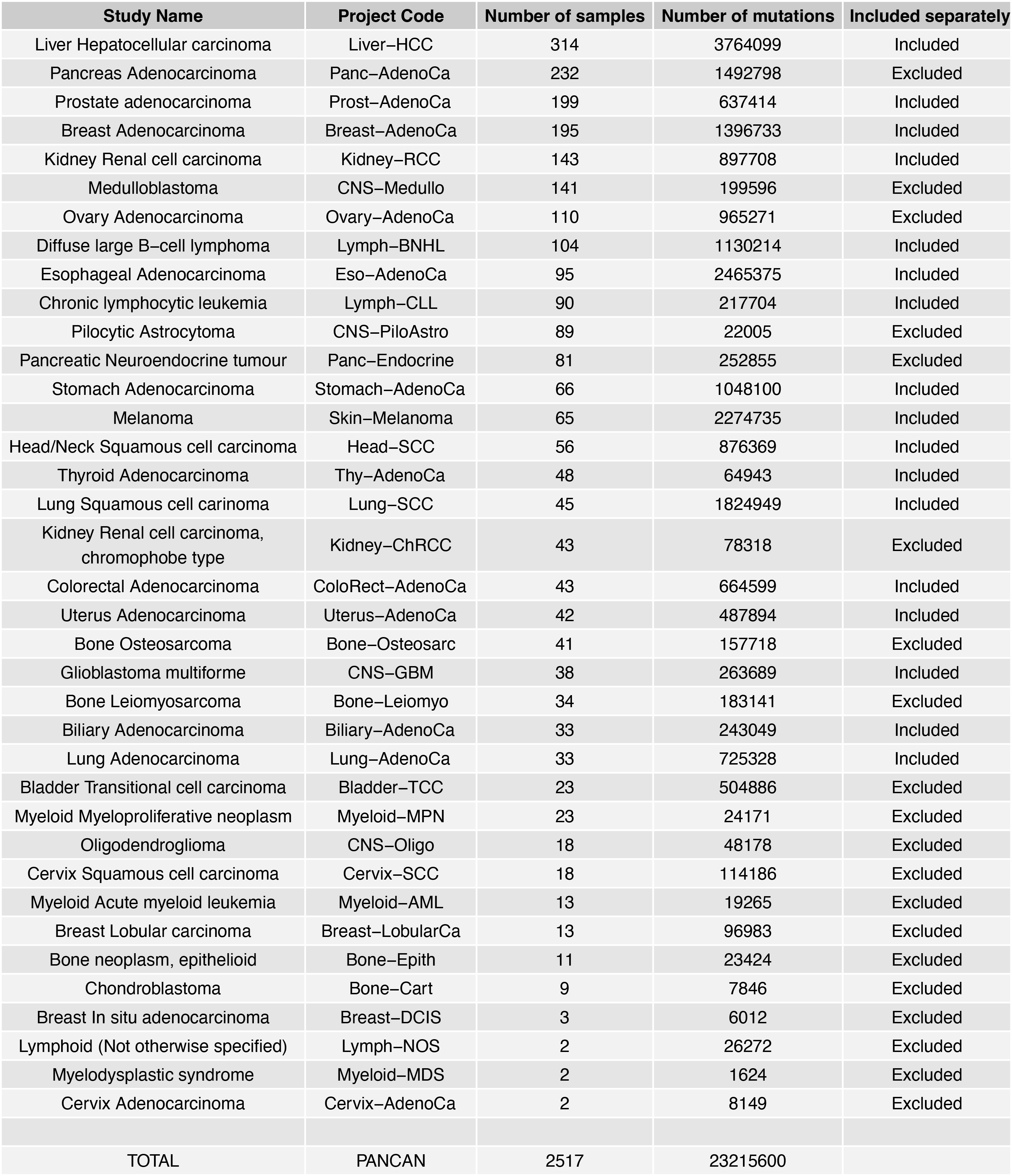

Supplement: S1 Fig — The rightmost column indicates whether the cancer type was included separately for regional mutation burden analysis. All cancer genomes were included in the pan-cancer analysis. (PNG) [file pcbi.1010393.s002.png]

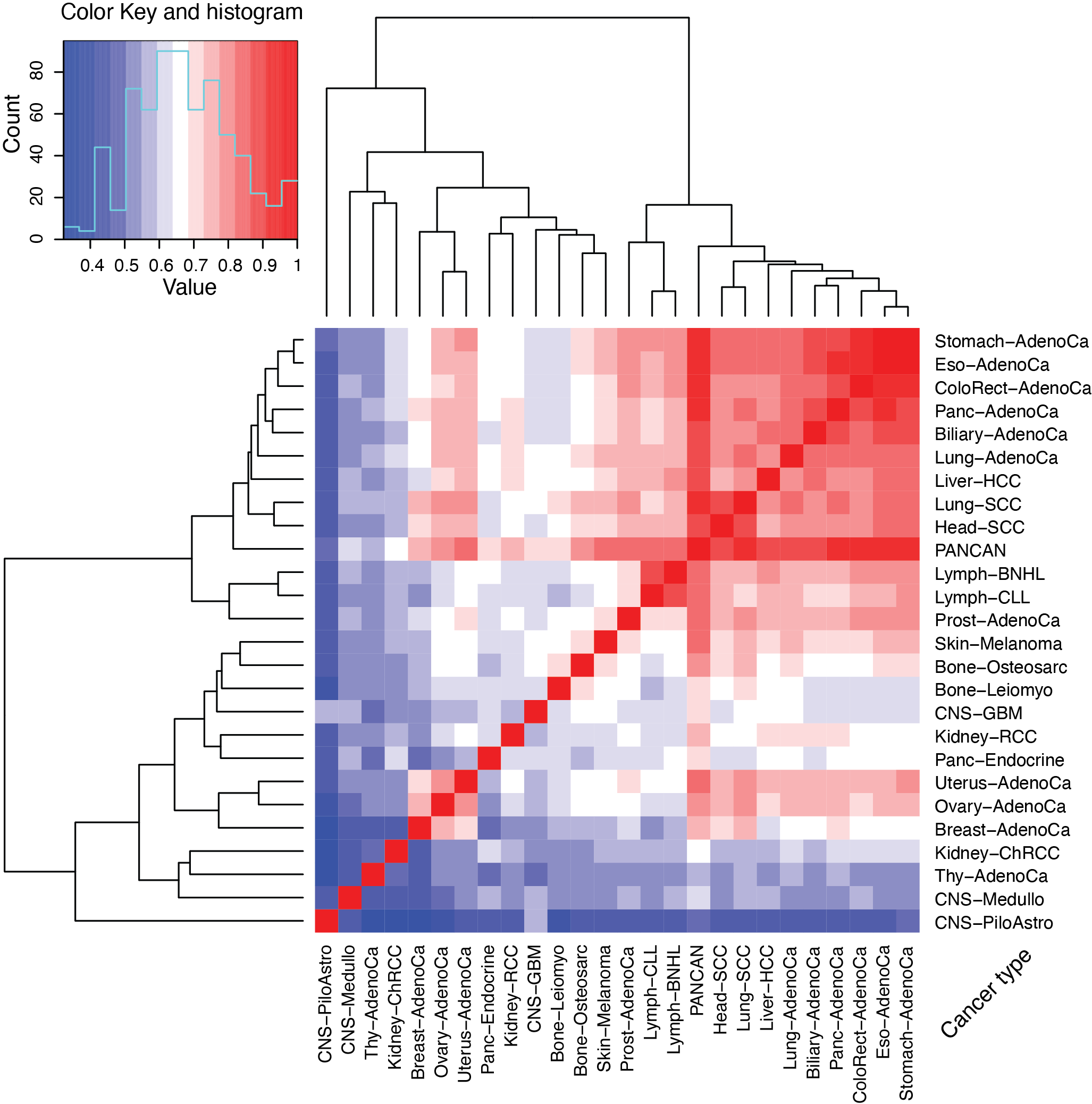

Supplement: S2 Fig — Clustering suggests similarity of cancer types in similar anatomical sites and cells of origin. For example, digestive tract cancers (stomach, esophageal, colorectal) and squamous cell cancers (lung, head & neck) are clustered. (PNG) [file pcbi.1010393.s003.png]

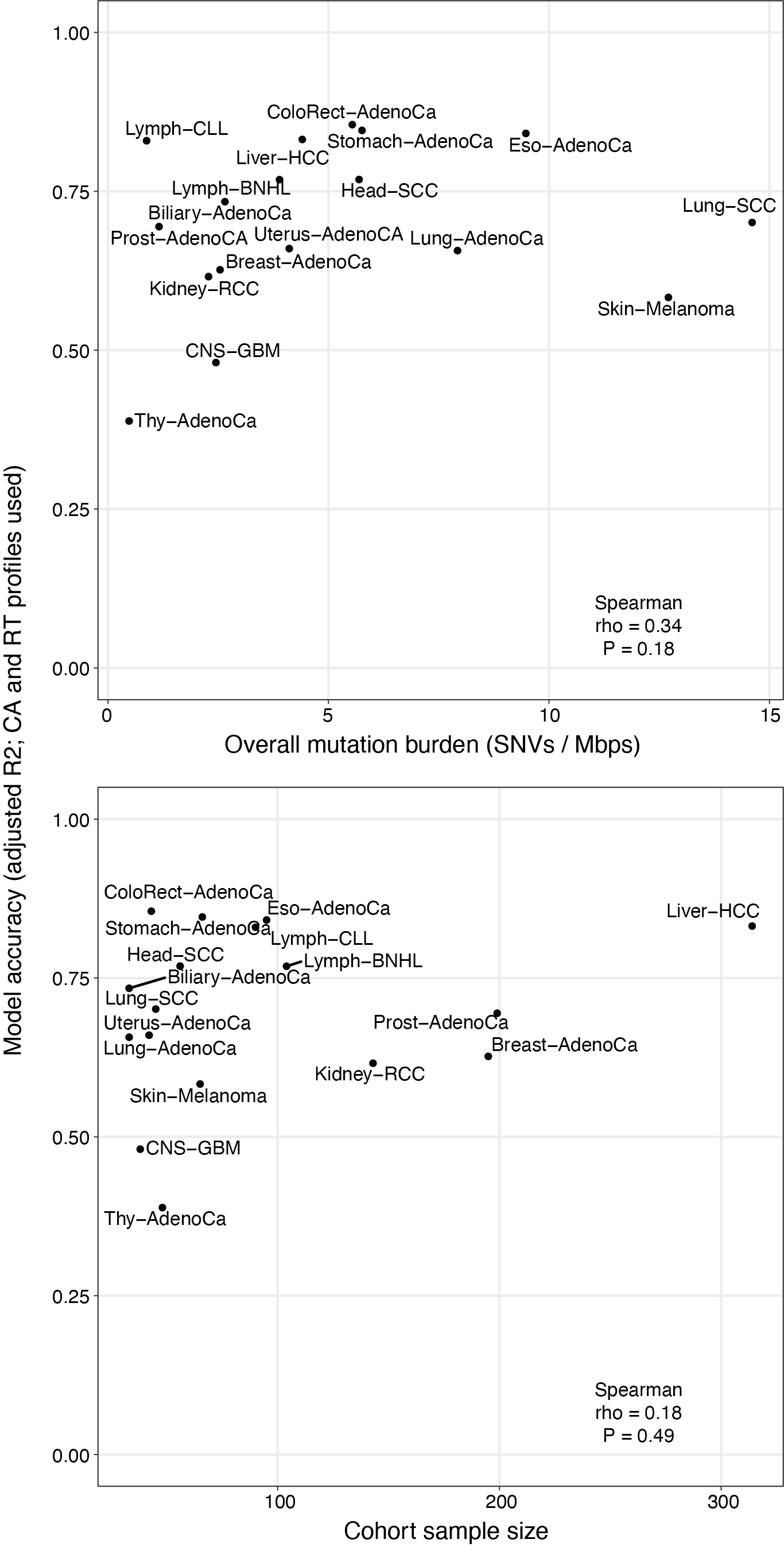

Supplement: S3 Fig — (PNG) [file pcbi.1010393.s004.png]

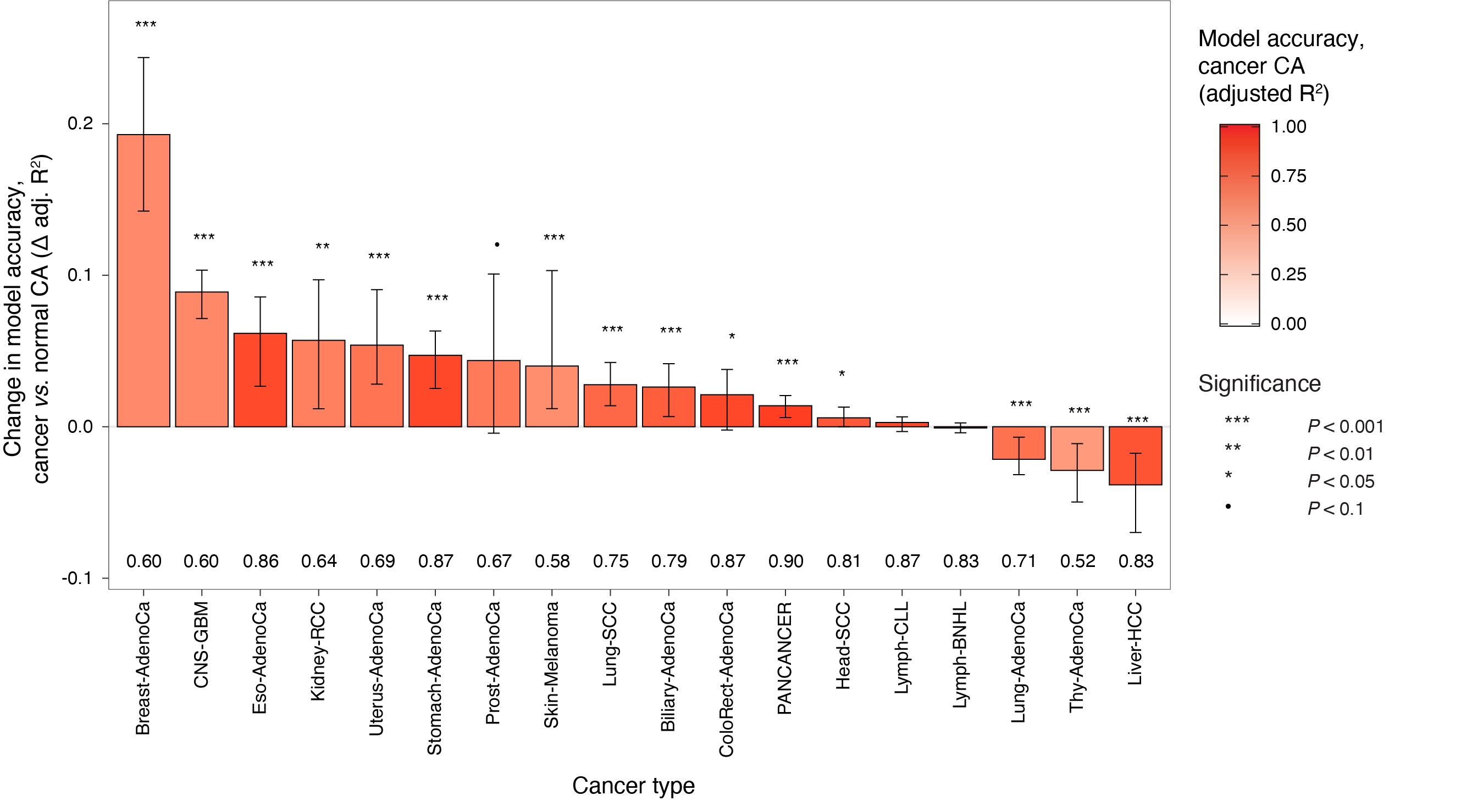

Supplement: S4 Fig — Bar plot shows the relative changes in prediction accuracy (Δ adjusted R2) of random forests informed by CA profiles of primary cancers, compared to matching models informed by CA of normal tissues. RT profiles are included in all models as reference. P-values of permutation tests and 95% confidence intervals from the bootstrap analysis are shown. Accuracy values of models informed by cancer CA profiles are listed below the bars (adjusted R2). (PNG) [file pcbi.1010393.s005.png]

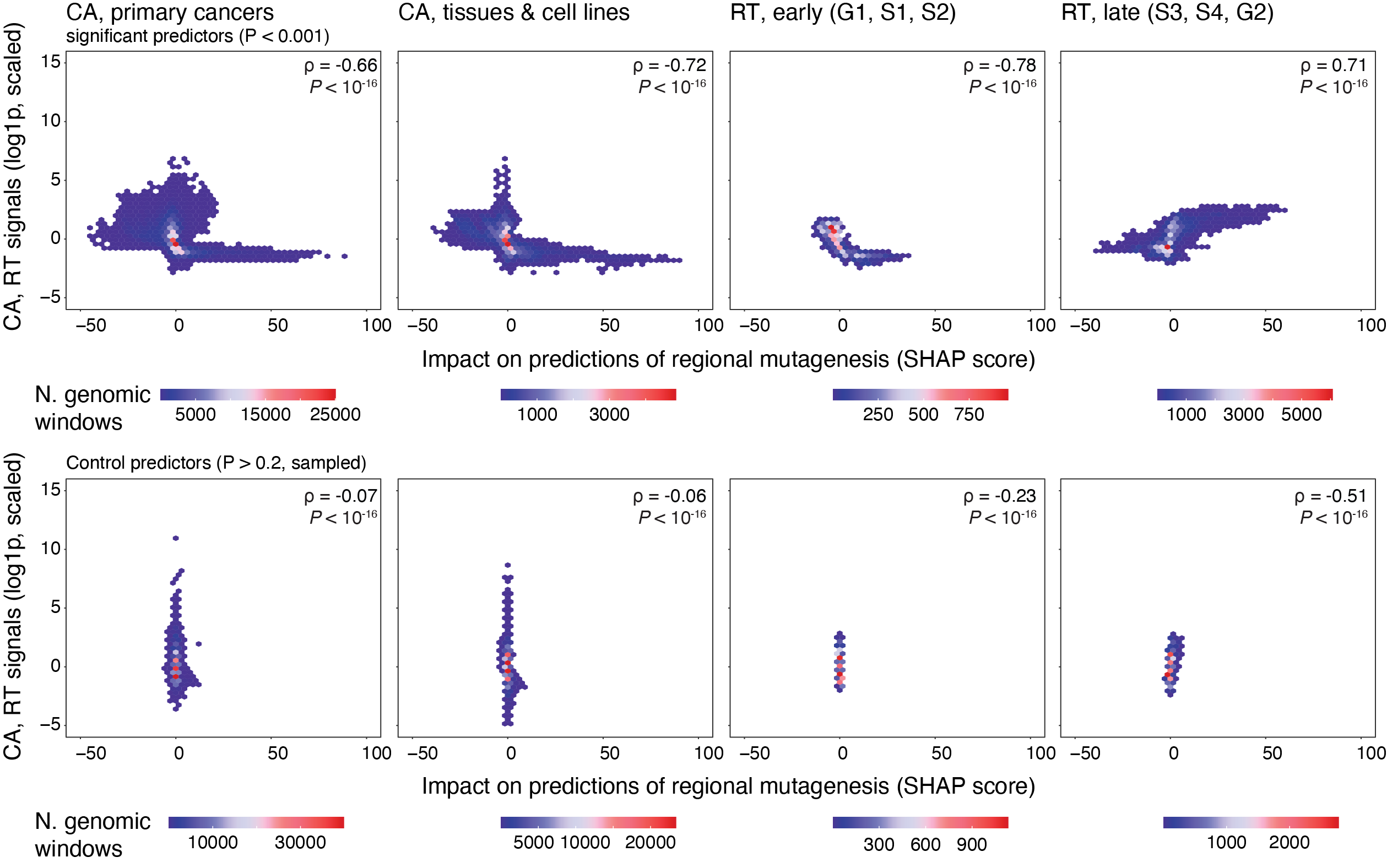

Supplement: S5 Fig — The top plots show the significant features across all cancer types (permutation P < 0.001). The bottom row shows non-significant features as controls (permutation P > 0.2). The non-significant features shown were sampled randomly from all non-significant features in equal numbers to significant features of individual cancer types. Spearman correlation coefficients and P-values are shown. (PNG) [file pcbi.1010393.s006.png]

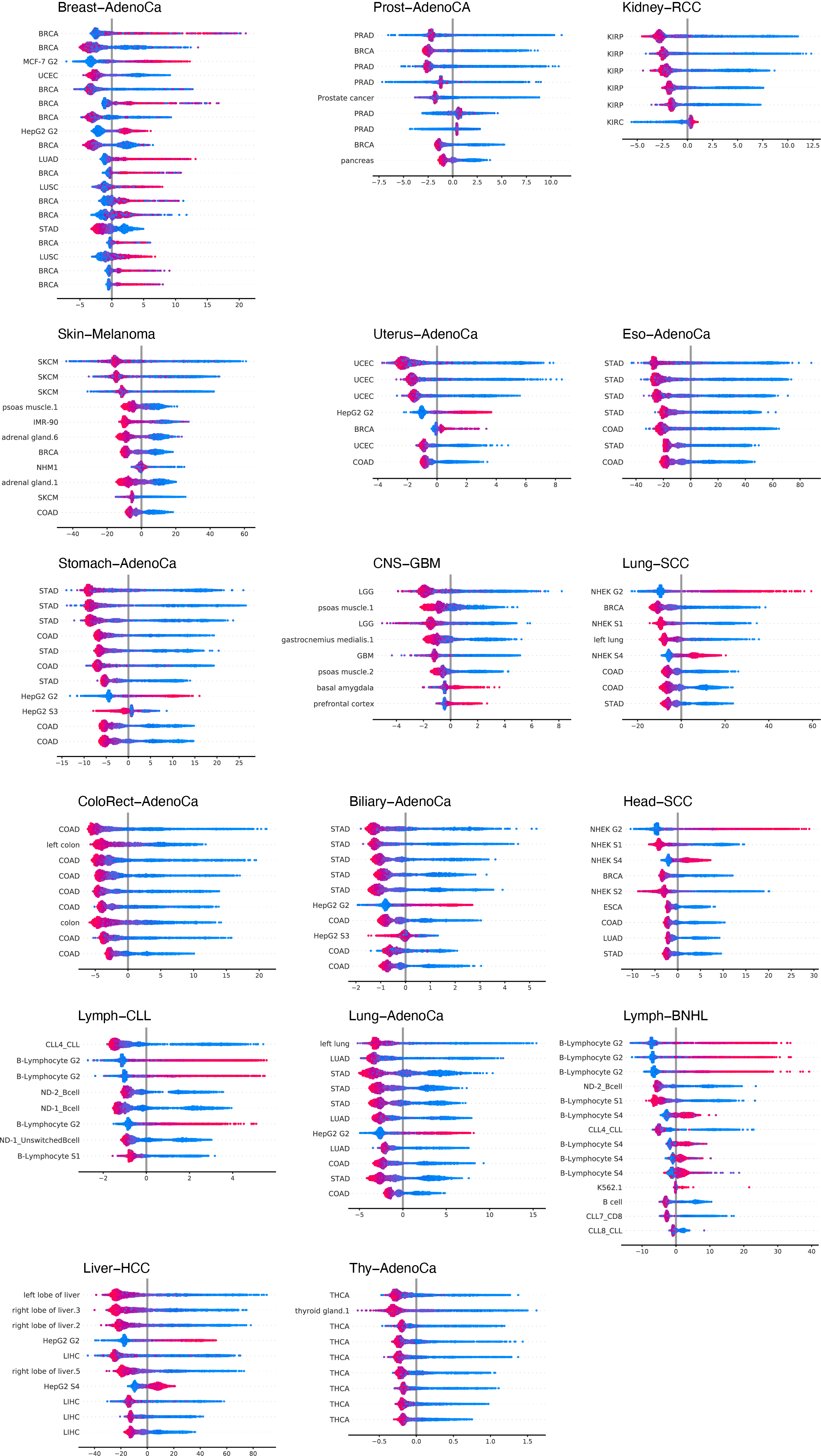

Supplement: S6 Fig — Significant predictors of megabase-scale SNV burden for all cancer types are shown (P < 0.001). Color shows CA or RT signal (blue, low; red; high) and X axis shows impact of CA/RT values on mutation rate predictions. (PNG) [file pcbi.1010393.s007.png]

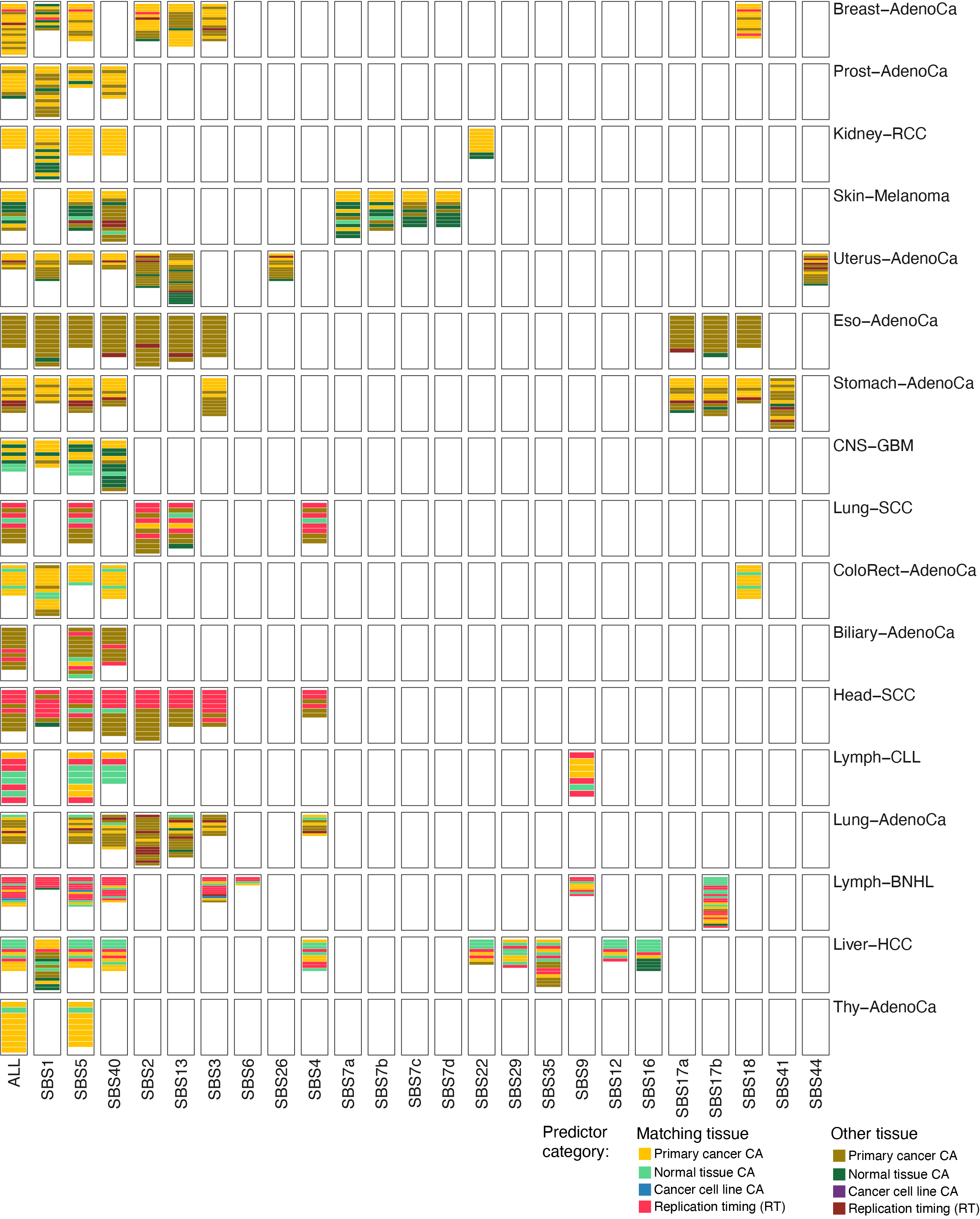

Supplement: S7 Fig — The first column shows the significant predictors of all SNVs. (PNG) [file pcbi.1010393.s008.png]

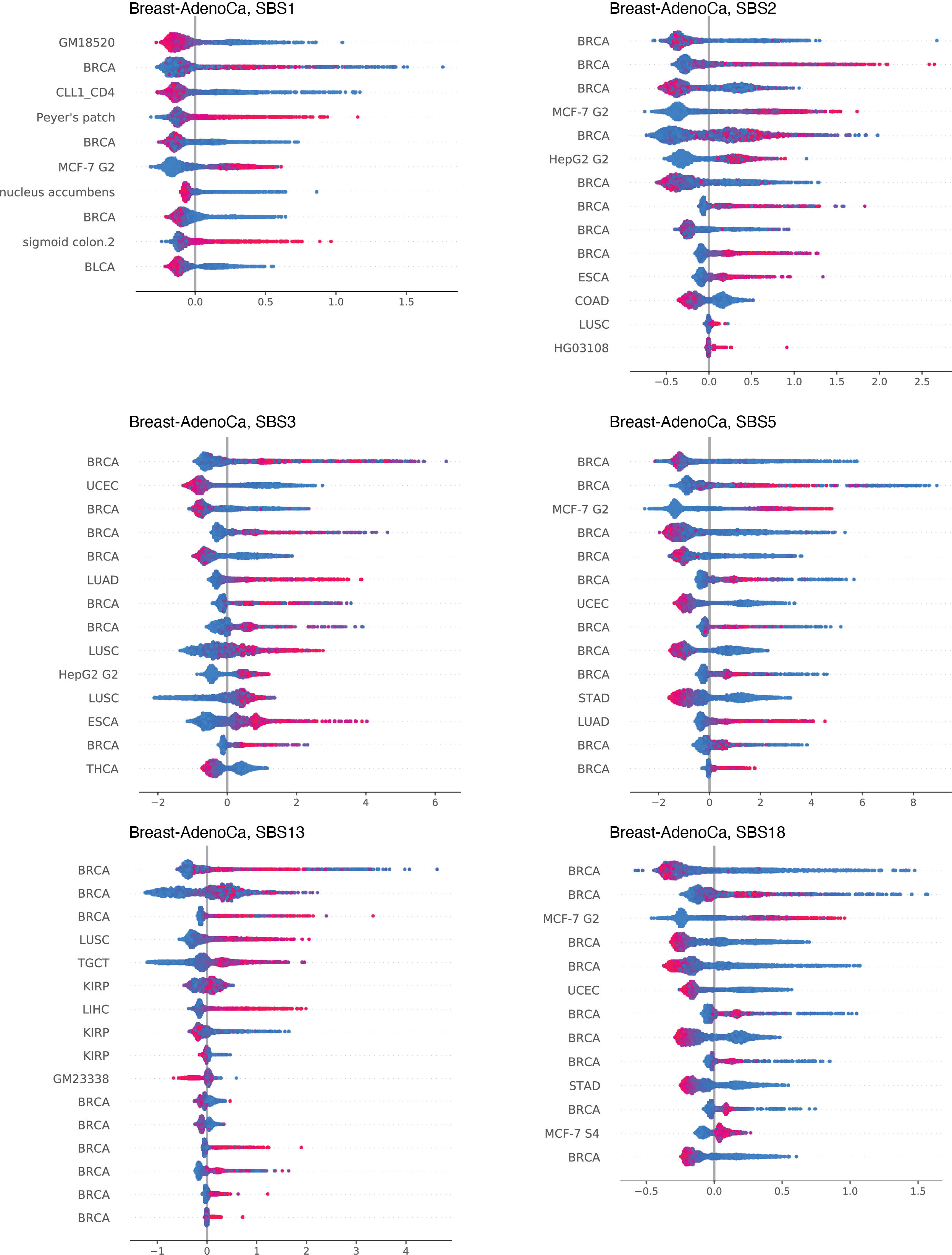

Supplement: S8 Fig — Significant predictors of megabase-scale burden of SNVs of different SBS signatures are shown (P < 0.001). Colors show CA or RT signal (blue, low; red; high) and the X-axis shows impact of CA/RT values on mutation rate predictions. (PNG) [file pcbi.1010393.s009.png]

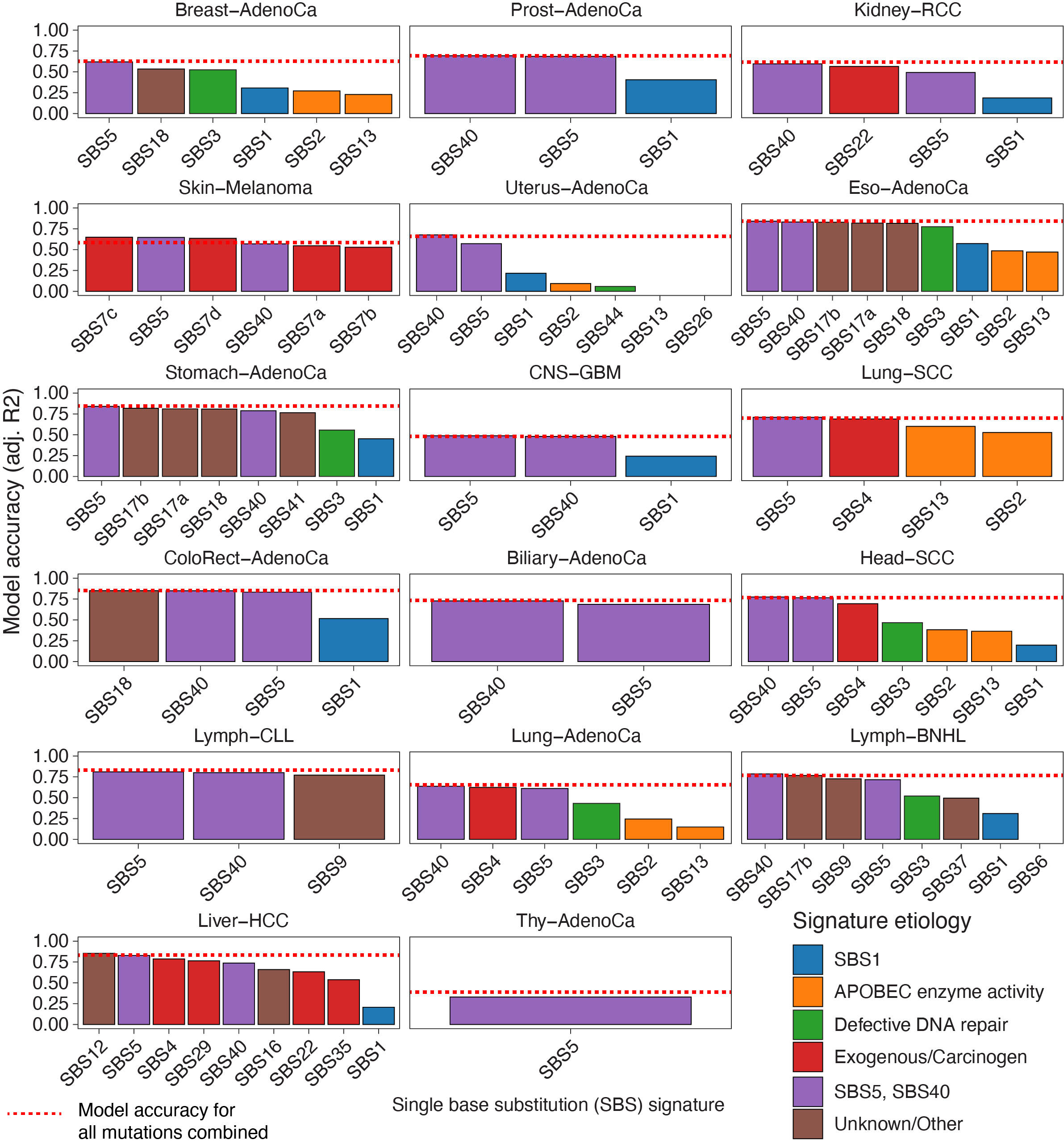

Supplement: S9 Fig — (PNG) [file pcbi.1010393.s010.png]

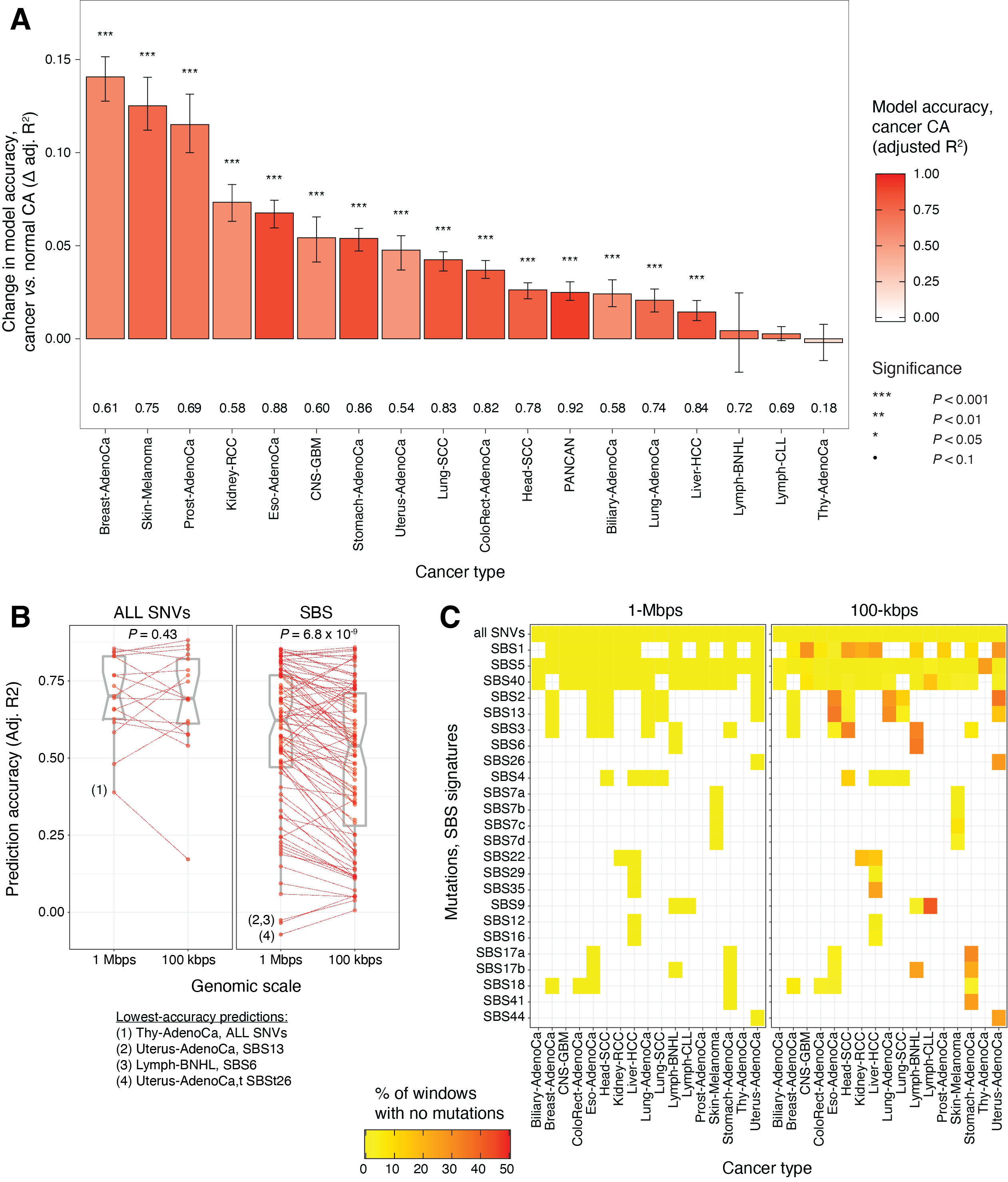

Supplement: S10 Fig — A. Improved prediction accuracy of cancer CA profiles is also observed at the finer resolution of 100-kbps genomic windows (see Fig 2A). B. Comparison of prediction accuracy of overall mutation burden (all SNVs, left) and SBS signatures (right). Paired Wilcoxon rank-sum P-values are shown. SBS predictions are significantly less accurate at the 100-kbps resolution. Examples of the least accurate mutation classes include the small thyroid cancer cohort that has a relatively low mutation burden, and infrequent SBS signatures for which many genomic windows have zero mutations. C. Heatmaps show the fractions of genomic windows with no mutations. Overall mutation burden (all SNVs) has few or no genomic windows with no mutations (top row), while the regional mutation burden of SBS signatures is less detectable at 100-kbps, and is seen in more windows with no mutations, especially in smaller cohorts and less-common SBS signatures. Data sparsity potentially explains the reduced prediction accuracy at the 100-kbps resolution. (PNG) [file pcbi.1010393.s011.png]

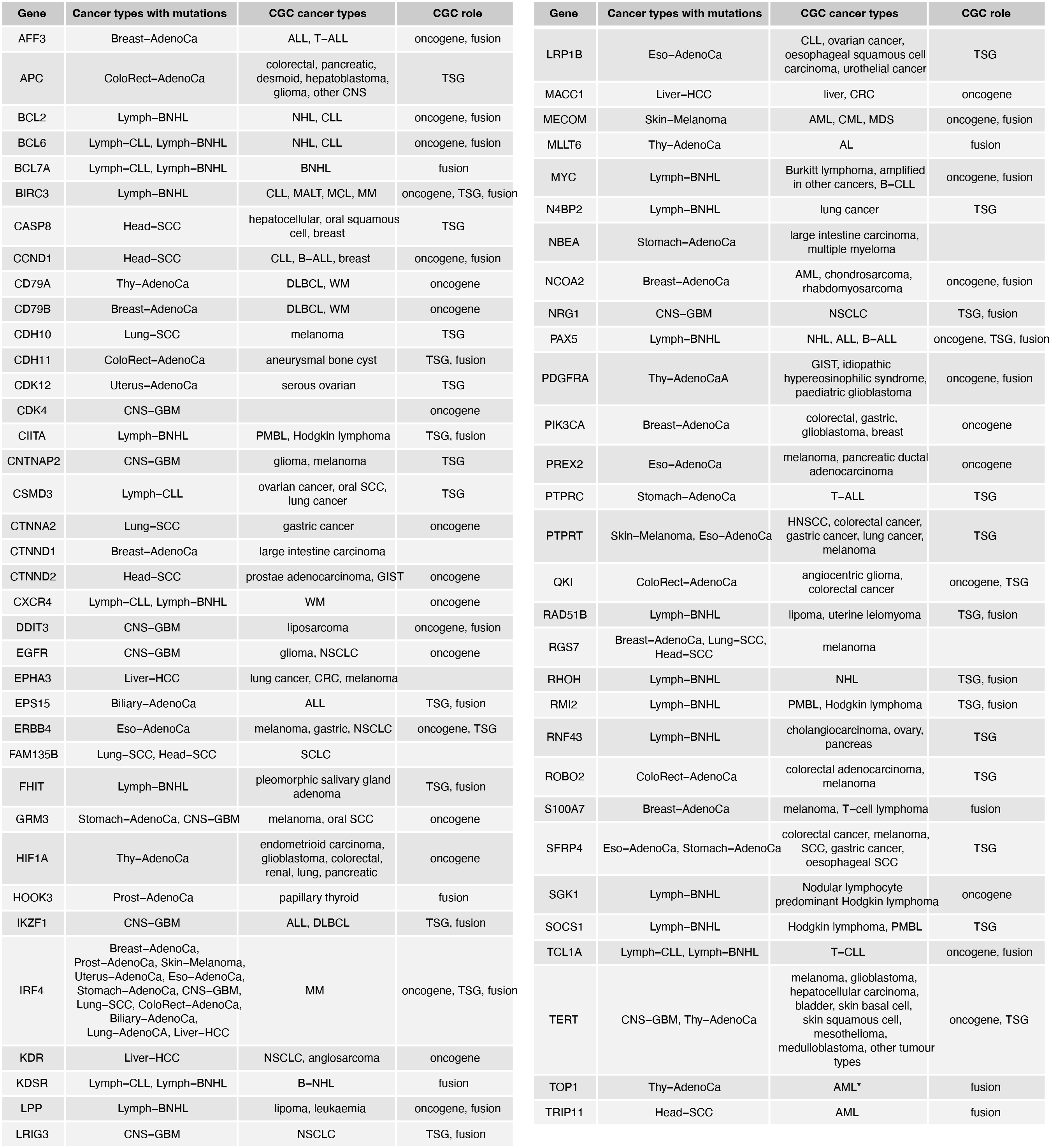

Supplement: S11 Fig — (PNG) [file pcbi.1010393.s012.png]

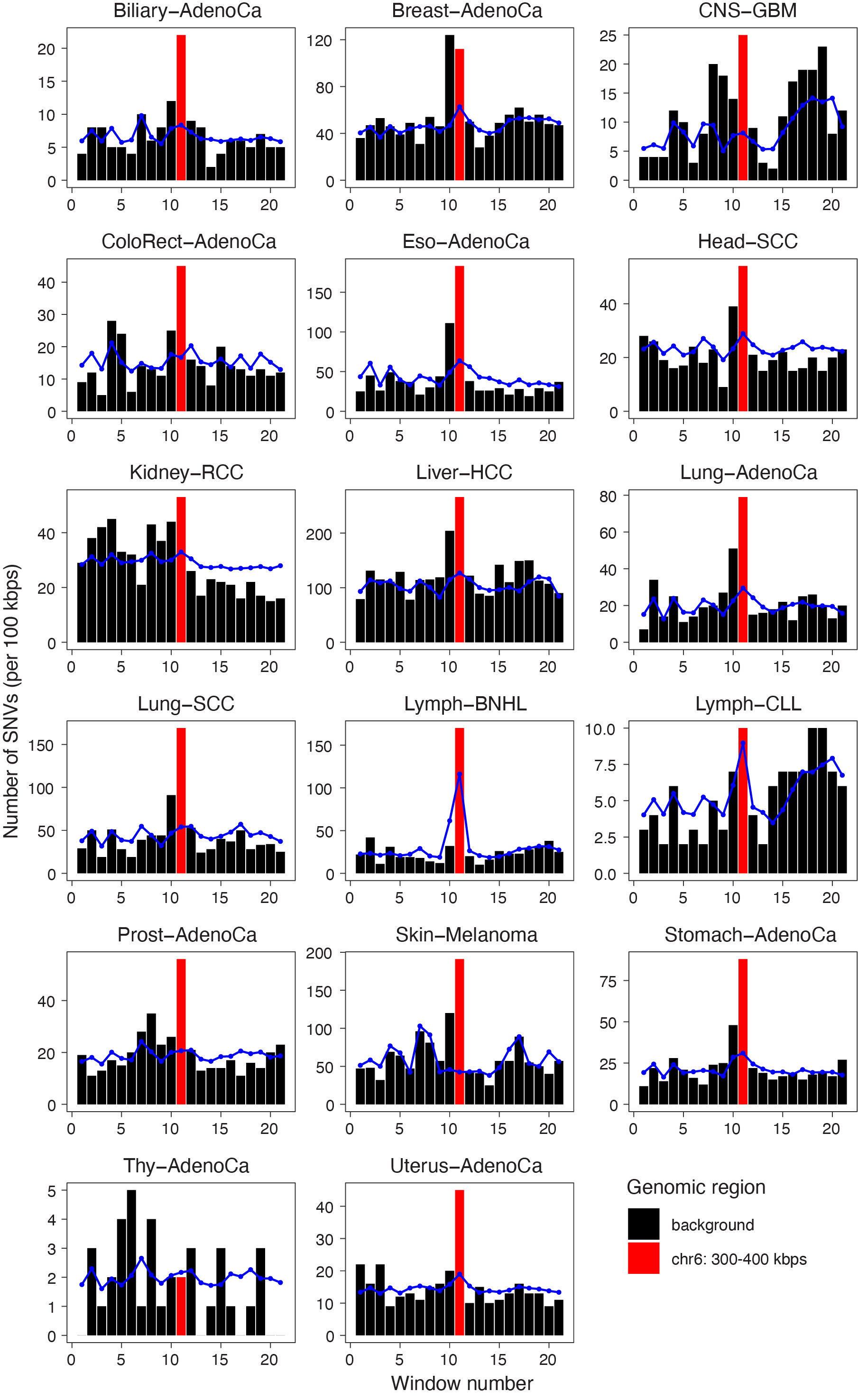

Supplement: S12 Fig — Bars represent 100-kbps regions. The predicted mutation burden from random forest models is shown in blue. (PNG) [file pcbi.1010393.s013.png]

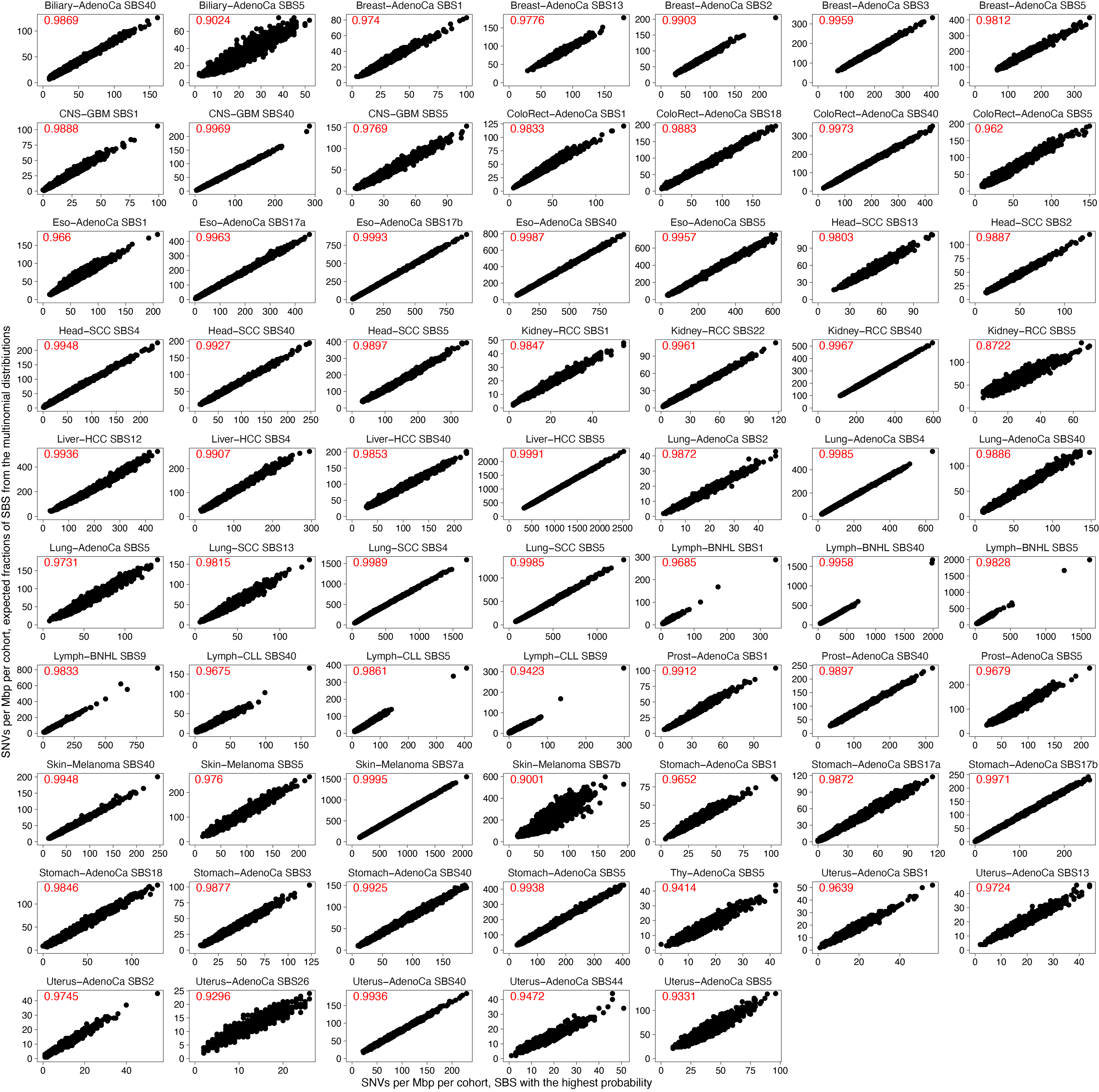

Supplement: S13 Fig — Scatter plots show the mutation counts of SBS signatures in cohorts of individual cancer types. The X-axis of each plot shows the SNVs assigned to the top-ranking signatures in each patient per 1-Mbp windows. The Y-axis shows the probabilistic assignment of all SNVs to all SBS signatures based on the expected values across the multinomial distributions of mutations in individual cancer genomes (as used in the study). Spearman correlation coefficients are shown in top-left corners of the scatter plots (all P < 10−16). Only sufficiently frequent SBS signatures were included (>20,000 SNVs per cohort based on both SBS annotation strategies). (PNG) [file pcbi.1010393.s014.png]
